# A comparative performance analysis of stand-alone, off-grid solar-powered sodium hypochlorite generators†

**DOI:** 10.1039/c9ra02221j

**Published:** 2019-05-08

**Authors:** E. Chinello, M. A. Modestino, J. W. Schüttauf, L. Coulot, M. Ackermann, F. Gerlich, A. Faes, D. Psaltis, C. Moser

**Affiliations:** School of Engineering, Ecole Polytechnique Fédérale de Lausanne (EPFL) Station 18 1015 Lausanne Switzerland enrico.chinello@epfl.ch +41 21 69 35171; Tandon School of Engineering, New York University (NYU) Rogers Halls 600A Brooklyn 11201 NY New York; Swiss Center for Electronics and Microtechnology (CSEM) Rue Jacquet-Droz 1 CH 2002 Neuchâtel Switzerland; Insolight SA Chemin de la Raye 13, Ecublens (VD) CH 1015 Lausanne Switzerland

## Abstract

Sodium hypochlorite (NaClO) is a chemical commodity widely employed as a disinfection agent in water treatment applications. Its production commonly follows electrochemical routes in an undivided reactor. Powering the process with photovoltaic (PV) electricity holds the potential to install stand-alone, independent generators and reduce the NaClO production cost. This study reports the comparative assessment of autonomous, solar-powered sodium hypochlorite generators employing different photovoltaic (PV) technologies: silicon hetero-junction (SHJ) and multi-junction (MJ) solar cells. For Si hetero-junctions, the series connection of either four or five SHJ (4SHJ and 5SHJ, respectively) cells was implemented to obtain the reaction potential required. MJ cells were illuminated by a novel planar solar concentrator that guarantees solar tracking with minimal linear displacements. The three solar-hypochlorite generators were tested under real atmospheric conditions, demonstrating solar-to-chemical conversion efficiencies (SCE) of 9.8% for 4SHJ, 14.2% for 5SHJ and 25.1% for MJ solar cells, respectively. Simulations based on weather databases allowed us to assess efficiencies throughout the entire model year and resulted in specific sodium hypochlorite yearly production rates between 7.2–28 g_NaClO_ cm^−2^ (referred to the PV surface), depending on the considered PV technology, location, and deployment of electronics converters. The economic viability and competitiveness of solar hypochlorite generators have been investigated and compared with an analog disinfection system deploying ultraviolet lamps. Our study demonstrates the feasibility of off-grid, solar-hypochlorite generators, and points towards the implementation of SHJ solar cells as a reliable technology for stand-alone solar-chemical devices.

## Abstract 2

Solar-powered electrochemical technologies can be employed to generate valuable chemical commodities on-site. We demonstrate solar-driven production of sodium hypochlorite, a widely employed water disinfection agent.

## Graphical abstract

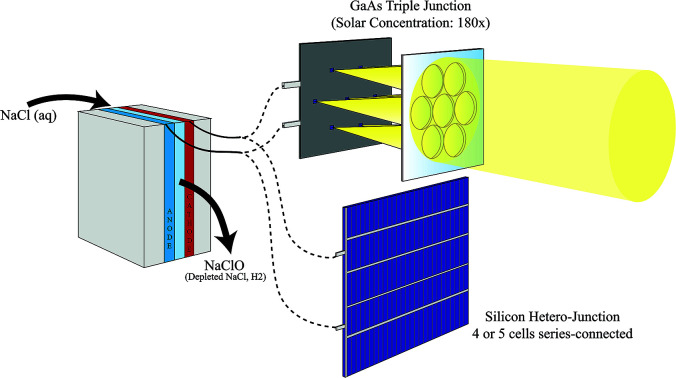

## 1. Introduction

The recent developments in solar technologies have paved the way for the autonomous operations of independent, off-grid installations, which employ renewable energy sources as primary feedstock. Beside the direct use to power appliances, illumination and auxiliary systems, photovoltaic (PV) electricity can drive electrochemical reactions and generate valuable chemicals, which can be transported and stored for later employment. Electrochemical processes are intrinsically inclined to PV integration, as they require direct electricity to operate; their scalability, modularity and ability to operate on-demand facilitates their stand-alone integration with renewable energy sources. Nevertheless, the intermittency of solar and wind power still poses major issues in the combination of renewables and large-scale electrochemical processes, which need to be operated continuously. A solar-powered, stand-alone, electrochemical device requires the following conditions to be met, in order to work at high efficiencies and represent an economic appealing alternative: (i) a discontinuous commodity demand (mitigated by storage, potentially); (ii) decoupling of PV source and electrochemical device – this route is likely more amenable for short-term implementation than a monolithic, fully integrated device;^1,2^ (iii) a comprehensive design of the components, whose dimensioning needs to be correlated and aimed at maximizing chemical throughput and conversion efficiency.^1,3^

Solar-electrochemical technologies are suitable and adaptable to numerous targeted outputs. Besides significant work on solar-powered hydrogen generators, little attentions has been devoted to the development of solar-electrochemical platforms for the generation of different commodities. Halides are of particular appeal as their principal production routes are electrochemical and they count on an established industrial practice.^4^ Chlorinated compounds are the simplest and most widespread means to eliminate organic contaminants contained in water streams or reservoirs.^5–8^ Sodium hypochlorite (NaClO) is a chemical commodity frequently employed for sanitation; its utilization range covers different sectors (*e.g.* water distribution, food industry, bleaching in other manufacturing processes).^9–11^ Sodium hypochlorite is typically generated *via* electrochemical routes in an undivided (*i.e.* membrane-less) reactor. The cell most commonly comprises a Dimensionally Stable Anode (DSA®, De Nora S.p.A.), to reduce overpotentials and maximize selectivity over the competing oxygen evolution.^12^ The main reaction by-product is hydrogen (H_2_), which could be further recovered and valorized to potentially sustain part of the process energy demands or impact different sectors (*e.g.* mobility). It is estimated that hydrogen recuperation could cover up to 15% of the process energy demand (if employed in a 50% efficient fuel cell). The possibility of generating sodium hypochlorite *in situ* and on-demand has gained significant attention over centralized production due to the elimination of transportation costs and for the superior germicidal properties of the solution.^10,13^

Tailored solar technologies are crucial to guarantee high conversion efficiency and ensure stability of operation in a broad range of working conditions. Several studies have attempted to deploy solar-electrochemical platforms to generate chlorinated compounds. Those reports coupled commercial silicon PV arrays to drive electro-chlorinators and sanitize water storages.^14–17^ These demonstrations were limited to <10% solar-to-chemical conversion efficiencies (SCE). Crystalline silicon (c-Si) based modules are appealing due to their low-cost, robustness and deployment ease; nevertheless, the limited output open circuit voltage (*V*_OC_) of c-Si solar cells (∼600 mV) requires the implementation of series-connected arrays to achieve the required potential to drive the electrolysis.^18^ c-Si hetero-junction (SHJ) technologies are attracting increased research and commercial interest.^19^ Their *V*_OC_ significantly exceeds 700 mV^20–22^ due to the excellent interface passivation provided by a thin layer (∼5 nm) of hydrogenated intrinsically amorphous-silicon (a-Si : H) between the c-Si and the oppositely doped layer.^23^ This allows arrays with fewer SHJ cells to drive electrochemical reactions, and therefore results in benefits from material savings and reduced module surface and cost. Multi-junction gallium-arsenide (MJ or GaAs) based technologies are currently emerging in the market and can potentially drive the electrochemical process directly, avoiding series-connection.^24,25^ Their implementation is consequently more appropriate for high-efficiency solutions. Given the high manufacturing and substrate cost, their use is tied to solar concentrator photovoltaics. The combination of multi-junction solar cells and solar concentrators has proven to be the means to achieve the highest reported SCE for a solar-driven electrochlorinator.^26^

**Fig. 1:**
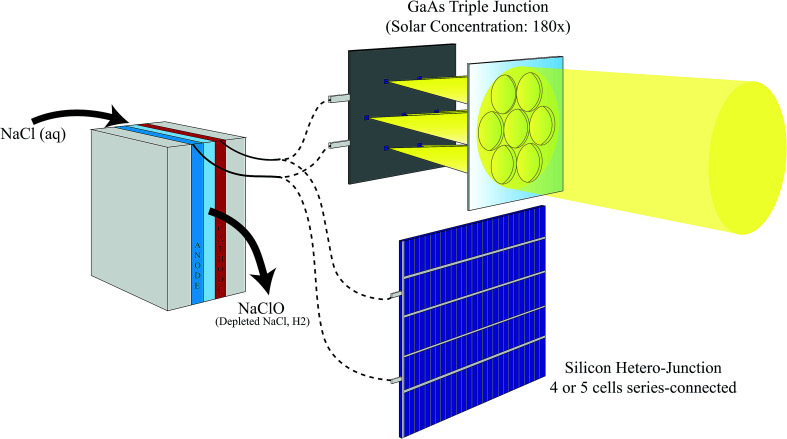
Schematic overview of a solar-powered sodium hypochlorite generator. The system is driven by silicon hetero-junction (series connection of multiple cells is required) or multi-junction photovoltaics. The latter needs to be illuminated by a solar concentrator in order to restrain the surface of the cells. Solar cells are deployed to operate a 3D-printed electrochemical cell, which converts a sodium chloride brine solution into a sodium hypochlorite stream. Hydrogen is generated as a by-product in the process.

In this study, we report a comparative analysis of solar-powered sodium hypochlorite generators employing two different PV technologies: (i) silicon hetero-junction or (ii) multi-junction GaAs solar cells under solar concentration (Fig. 1). SHJ mini-modules comprised either four or five cells connected in series (4SHJ or 5SHJ, respectively), whereas the multi-junction solar cells were illuminated using a novel planar solar concentrator, which ensures solar tracking with minimal linear displacements of the optics. Outdoor testing under real atmospheric conditions revealed maximum SCE of ∼10%, ∼14% and ∼25% for the devices powered by 4SHJ, 5SHJ and MJ cells, respectively. The discrepancies in conversion yields corresponded to different operative conditions of the solar-hypochlorite generators. Simulations considering three different locations assessed the potential annual sodium hypochlorite productivity for the PV technologies.

To the best of our knowledge, this is the first experimental and computational comparative analysis of solar-driven sodium hypochlorite generators employing high efficiency silicon-based and multi-junction PV technologies.

We analyzed the potential deployment of solar-hypochlorite solutions to sanitize organic contaminants in remote and isolated locations, where water effluents most likely need treatments against waterborne pathogens. The guidelines we provide supply useful insights for design and operating stand-alone, independent, off-grid solar-NaClO devices. The technology we have analyzed has the potential to spur further innovation in the water disinfection domain and contribute to supply access to potable drinking water for communities in need.^27–30^

## 2. Materials and methods

The tested devices comprised two main components: (i) a photovoltaic element, illuminated by a planar solar concentrator in the case of multi-junction solar cells; (ii) an electrochemical reactor fabricated *via* additive manufacturing to carry out state-of-the-art brine electrolysis. The prototypes were tested outdoors under natural sunlight to assess the conversion efficiencies and indoors to evaluate their stability over prolonged illumination.

### 2.1 Silicon hetero-junction solar cells and modules

Silicon hetero-junction solar cell precursors were obtained from Chochu Industry Corp. and consisted of textured monocrystalline silicon wafers. These wafers were coated with hydrogenated amorphous silicon buffer and doped layers by plasma-enhanced chemical vapor deposition. Subsequently, thin transparent conductive oxide layers were sputtered on the samples. Metallization was applied on the cell precursors by screen-printing of low curing temperature silver paste with a 4 busbar design and cured at 210 °C for 30 min. Full wafers were laser cut into 4 sub-cells. Cell interconnection was performed using the so-called “shingling” approach using silver electrical conductive adhesive to contact one cell to the adjacent one. The module design was based on a glass–glass configuration using poly-olefin as encapsulant. The surfaces of the SHJ minimodules were partially covered using masks, which left an exposed area of 43.3 and 53.4 cm^2^ for the 4SHJ and 5SHJ modules, respectively.

### 2.2 Multi-junction solar cells and solar concentrator

The prototype powered by MJ PV employed seven solar cells (0.6 × 0.6 mm^2^) in parallel. The cells were a triple-junction stack InGaP/GaAs/InGaAsNSb (bandgaps 1.9/1.4/1.0 eV) and were mounted on a printed circuit board (Fig. S1 in the ESI†).

The novel planar solar concentrator was developed by Insolight SA (Ecublens, Switzerland) and exploits minimal linear movements of the concentrator optics (<5 mm) to track the sun. It relies on an innovative optical design of the lens array. Each lens is bi-convex and radially-symmetric; it was modelled through a free-form design process to flatten the Petzval curvature field. A prototype of seven custom-shaped lenses, hexagonally disposed, was fabricated in moldable silicone (MS-1002) injected in an aluminum mold. In normal incidence conditions, the working focal length was approximately 6 mm. The design concentration factor was 180×. The optical efficiency of the solar concentrator was certified by an independent institution (Fraunhofer ISE, Freiburg, Germany) to be as high as 80% even with a 40° tilted illumination.

### 2.3 Electrodes and electrolysis cell

Electrodes were purchased from Industrie De Nora S.p.A. (Milan, Italy). The sheets consisted of a titanium substrate (1 mm thick) coated with a mixed metallic oxide blend – MMO (DSA®). We employed identical electrocatalysts for the anodic and cathodic reactions (eqn (1) and (2), respectively), as often done in industrial practice. The electrode effective surface was 2.2 cm^2^ (*∅* 17 mm).

1 2Cl^−^ → Cl_2_ + 2e^−^, *E*^0^ = 1.3583 V_SHE_

2 2H_2_O + 2e^−^ → H_2_ + 2OH^−^, *E*^0^ = −0.8277 V_SHE_

3 2NaCl + 2H_2_O → Cl_2_ + H_2_ + 2NaOH, *E*^0^_id_ = 2.186 V_SHE_

Eqn (3) represents the overall, unperturbed reaction, obtained by addition of the two half reactions. *E*^0^_id_ is the cell potential in standard conditions and is the value of choice for all further calculations; this permits to compare devices even in case they are operated in different working conditions. Chlorine species can undergo further chemical transformations, depending on the working conditions of choice for the electrolyte.^31^

4 Cl_2_ + H_2_O ⇌ H^+^ + HOCl + Cl^−^

5 HOCl + H_2_O ⇌ ClO^−^ + H_3_O^+^

Calculations based on the equilibrium constants revealed that Cl_2_, HOCl and ClO^−^ species are predominant at very acidic, slightly acidic/neutral and basic pH values, respectively.^31,32^ It is therefore desirable to have an electrolyte pH > 10 in order to shift the reaction equilibrium towards hypochlorite ions over competing species. Despite multiple reaction mechanisms could potentially lead to hypochlorite species formation (*e.g.* the competitive direct oxidation of chloride to hypochlorite), the eqn (3)–(5) pathway is generally the predominant in the working conditions of choice.^31,32^ The basic pH of NaClO solutions is compatible with drinking water treatment (*i.e.* neutral pH), as the required dosage is in the order of mg L^−1^; thus, it does not significantly affect the final pH. The electrolyte was a 20% wt sodium chloride solution, whose pH was adjusted with the addition of sodium hydroxide; it was prepared dissolving high-purity NaCl (>99.5%) purchased from Roth GmbH, in de-ionized water and adding the desired quantity of NaOH (Reactor Labs SA). The pH (∼11) was monitored using a VWR pH110 pH-meter, calibrated using Merck Certipur® buffer solutions – pH 4.01/7.00/10.01.

Two identical flow plates were designed to hold the electrodes and supply/extract the charges necessary to sustain the reaction; the plates were 3D-printed in stainless steel and electroplated in gold by Shapeways Inc. (NY, U.S.). The utilization of robust materials was necessary to ensure resistance over chlorine and electrolyte corrosion. An additional separator plate was fabricated by VeroWhite, employing additive manufacturing (Stratasys, Object500 Connex); this piece ensured the correct inter-electrode working distance (3 mm). Sealing was guaranteed through nitrile rubber (NBR) O-rings. A schematic overview of the electrochemical cell is depicted in Fig. S2 in the ESI.†

Ion-exchange membranes are not desirable in hypochlorite cells, since no product separation is necessary, and they limit the chlorine downstream reactions (eqn (4) and (5)). Nevertheless, this allows hypochlorite species (ClO^−^) to be reduced at the cathode; thus, faradaic efficiencies (*η*_Faraday_) are generally lower for hypochlorite production (*i.e.* membrane-less cell) than for chlorine gas generation (*i.e.* membrane-based cell).^31^

The electrolyte was circulated in a closed loop *via* a peristaltic pump (New Era Pump Systems Inc. NE 9000) at a 40 mL min^−1^ flow rate. Electrolyte replenishment was not performed. The solution was maintained and monitored at the working temperature of 80 °C (batch temperature) using a VWR hotplate (VMS C-7) and a 10′′ steel temperature probe (VWR International) immersed in the continuously stirred electrolyte. It is worth underlining that a stand-alone solar-hypochlorite device would need to comprise a solar-heater, as the reaction kinetics degrade considerably with lower operation temperatures.

Electrochemical characterizations and measurements were performed using a Bio-logic potentiostat VSP-300 at 20 mV s^−1^ scan-rate.

### 2.4 Outdoor testing

**Fig. 2:**
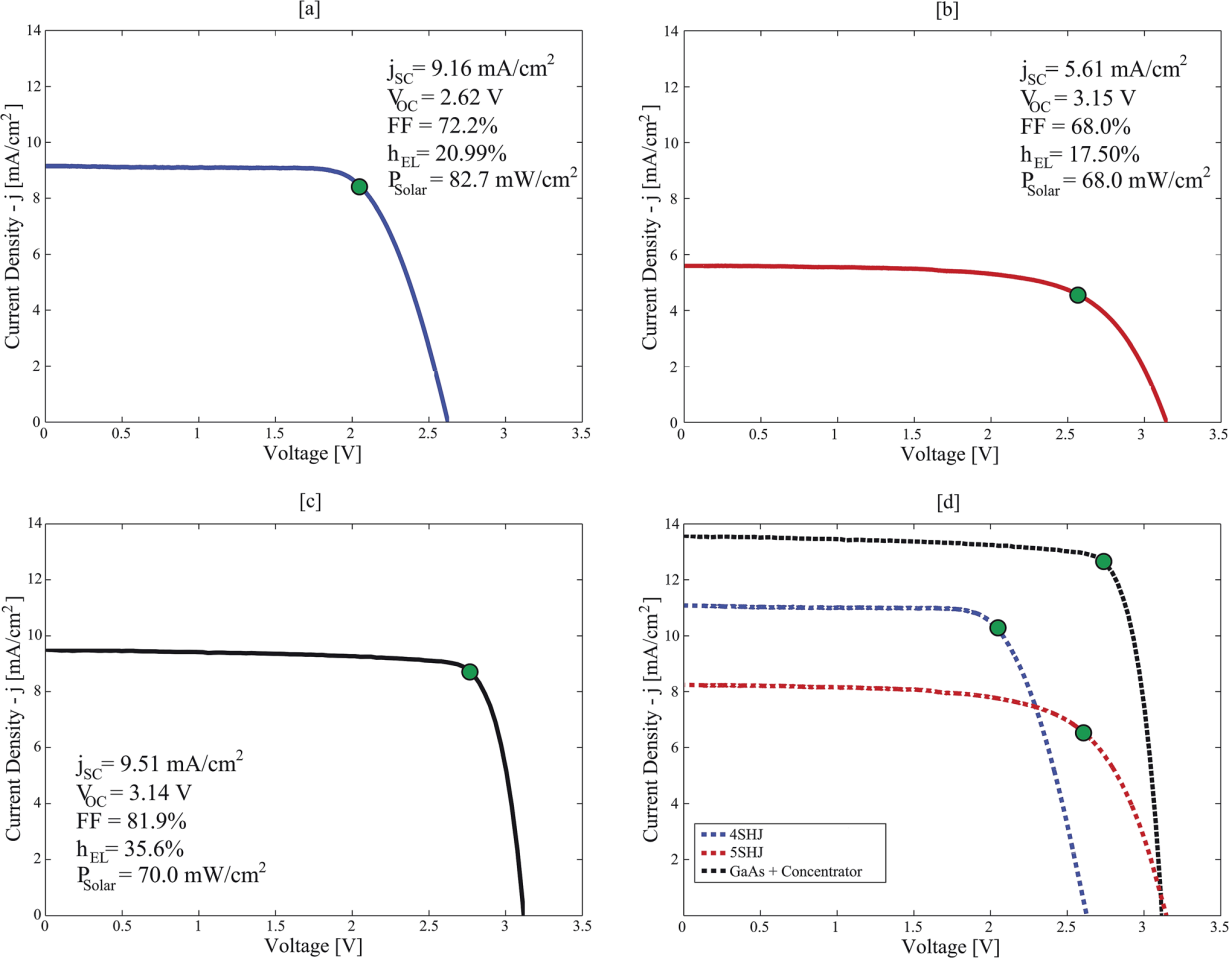
(a): *j*–*V* curve characterization of a 4SHJ minimodule under real atmospheric conditions, 26^th^ June 2017, Lausanne (CH). (b): *j*–*V* curve characterization of a 5SHJ minimodule under real atmospheric conditions, 26^th^ June 2017, Lausanne (CH). (c): *j*–*V* curve characterization of multi-junction GaAs-based solar cells illuminated *via* Insolight solar concentrator under real atmospheric conditions, 6^th^ August 2017, Lausanne (CH). (d): scale-up *j*–*V* characteristic curves at 1000 W m^−2^ solar power input. Green dots mark the position of the MPPs.

The solar-hypochlorite devices were tested under real atmospheric conditions on 26^th^ June and 5^th^ July 2017, at the EPFL campus in Lausanne, Switzerland (46°31′13.9′′N – 6°33′54.8′′E). The 4SHJ minimodule was positioned horizontally and tested under real solar illumination on 26^th^ June between 13–15 h (Fig. 2a). The 5SHJ minimodule, aligned horizontally, was tested on 26^th^ June between 11–13 h (Fig. 2b). The electrochemical device powered by MJ cells was evaluated on 5^th^ July 2017, between 12–14 h (Fig. 2c); initially tilted at 60° and aligned along the 130°SE direction, the linear tracking was periodically manually adjusted to maintain high current throughput and efficiency (Fig. S3 and S4 in the ESI†). Solar irradiance data were retrieved from the Solar Energy and Building Physics Laboratory (LESO-LB, EPFL) online database – https://leso2.epfl.ch/eibknx. Global and direct irradiance on the horizontal (GHI and DHI, respectively) data were obtained and utilized to calculate the direct normal irradiance (DNI) given the exposure angle of the solar concentrator input aperture.

### 2.5 Indoor testing

A ScienceTech SF-300 class AAA solar simulator was employed to carry out the device stability tests under intermittent and continuous exposure. The solar simulator spectral characteristics are reported in Fig. S5 in the ESI.†

In order to assess the device sensitivity to intermittent illumination, we exposed the PV cells to the solar simulator beam for 180 seconds, alternating 180 seconds of light with 180 seconds of darkness.

We continuously illuminated the solar cells for 100 hours to evaluate the performance degradation.

### 2.6 Faradaic efficiency calculation

The current efficiencies of the solar hypochlorite devices were assessed indoors. The PV modules were illuminated by means of the ScienceTech solar simulator, whose power was adjusted to obtain two different amperages at each electrode surface: 300 mA (∼130 mA cm^−2^, with respect to the electrode surface) for SHJ modules and 60 mA (∼30 mA cm^−2^, with respect to the electrode surface) for MJ solar cells illuminated by the solar concentrator. The two different conditions were intended to recreate the average outdoor illumination conditions.

The electrolyte was circulated at 40 mL min^−1^ by the peristaltic pump in a closed loop comprising a buffer volume, from which samples were periodically collected to be analyzed. The amount of sodium hypochlorite was estimated using a Lovibond® CHECKIT colorimetric comparator test-kit for high range (10–300 mg L^−1^ of product).

Faradaic efficiencies were calculated by comparing the experimental measurements to the theoretical values. Results showed *η*_Faraday_ of 88% and 85% for 130 mA cm^−2^ and 30 mA cm^−2^, respectively (Fig. S6 in the ESI†). Those values were consistent with previous reports.^33,34^ The non-ideality of faradaic efficiencies relies on two main mechanisms: (i) the competing oxygen evolution, due to the abundance of hydroxyl ions in solution, which is favored by high pH values but counteracted by the selectivity of the anodic coating;^35^ (ii) the diffusion of oxidized hypochlorite species at the cathode, where they are back-reduced to chloride species, which could be restrained by introduction of external compounds (*e.g.* sodium dichromate^36,37^).

## 3. Results and discussion

### 3.1 Experimental demonstration

Three solar-hypochlorite devices were tested outdoors under real solar illumination, powered by 4SHJ solar cells, 5SHJ solar cells and MJ solar cells illuminated by a solar concentrator, respectively. In the latter configuration, we utilized a novel technology developed by Insolight SA that employs millimetric linear displacements (±5 mm) to track the sun over a broad angular range. The 36.4% electrical efficiency of a seven lens array prototype illuminating MJ cells was certified by Fraunhofer ISE (Freiburg, Germany), as well as the angular efficiency (*η*_optical_ = 80% at ±40° illumination tilt). Due to the short focal length and linear tracking needed, it is expected that this concentrator can be integrated in flat panels with thickness similar to those commercially available for rooftop modules. Its single-lens design guarantees superior light transmission properties than multi-element configurations commonly used for planar concentrators^38,39^ (*i.e.* transmission losses < 8%). Furthermore, the technology can be scaled and implemented in m^2^-scale arrays.

**Fig. 3:**
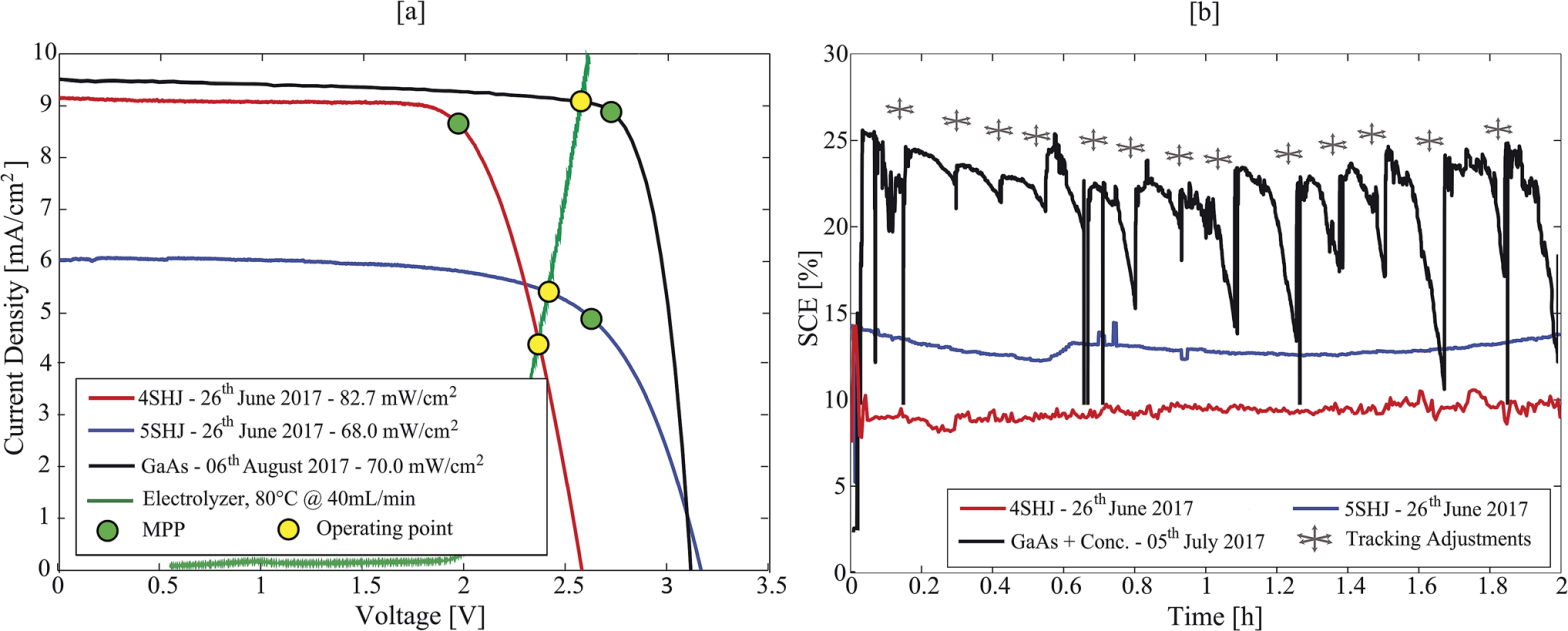
(a) Experimental *j*–*V* curves for the three different PV technologies; MPPs and working points are reported to highlight their relative positions. (b) Experimental SCEs for the three different technologies; data for 4SHJ-devices were obtained on 26^th^ June 2017, 1–3 PM, data for 5SHJ-devices were obtained on 26^th^ June 2017, 11 AM – 1 PM, data for MJ-devices were obtained on 5^th^ July 2017, 10 AM – 12 PM.

In order to ensure high conversion yields, the PVs and the electrochemical cell have to be effectively current-matched (Fig. 3a). By using working potentials (*V*_OP_) lower than the maximum power point voltage (*V*_MPP_), this ensures stability of operation and high solar-to-chemical efficiency (SCE) throughput (eqn (6)).^1^6
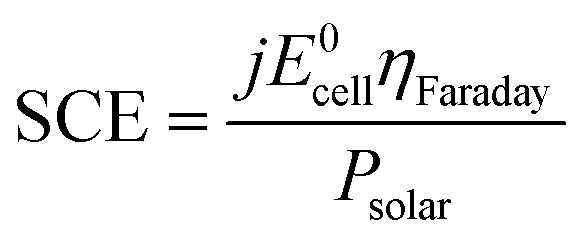
where *j* is the measured current density, in mA cm^−2^; *E*^0^_cell_ the cell working potential (2.188 V), given the negligible effect of the mixing contribution;^26^*η*_Faraday_ the cell faradaic efficiency; *P*_solar_ the solar power input (GHI for 4SHJ and 5SHJ; DNI for MJ cells coupled with concentration), in mW cm^−2^, retrieved from the LESO-SB weather station online platform. It was estimated that the confidence interval for the experimental SCEs was ±7.1%.

The 4SHJ minimodule did not satisfy the *V*_OP_ requirement, as the operating point is located at potentials higher than *V*_MPP_. Therefore, the solar-hypochlorite device employing this technology showed the lowest and most fluctuating SCE (8.2–10.2%) (Fig. 3b). The stability condition was fulfilled by the devices comprising 5SHJ and MJ cells under solar concentration; we recorded SCEs within 13.7–14.9% and up to 25.2%, respectively (Fig. 3b and S3 in the ESI†). The latter findings are consistent with other studies previously reported employing GaAs solar cells and solar concentration.^26^ It is worth stressing that for a PV technology to ensure stability of operation, it has to fulfil the condition: *V*_MPP_ > *E*^0^_cell_ + *η*, where *η* corresponds to the reaction overpotentials. In order to maximize the SCE throughput, the electrochemical cell must then be tailored to target the current-load match and to ensure a *V*_OP_ lower than *V*_MPP_.

### 3.2 Computational assessment of solar-hypochlorite devices in different climates

Computational analyses based on weather data were conducted in order to assess the SCE and potential hypochlorite productivity of the solar-powered devices in three different locations: Lausanne (Switzerland), Phoenix (U.S.) and Delhi (India) (Table 1). In the simulations, PVs were oriented facing the equator. For each case, two situations were simulated: (i) fixed PV tilt angle throughout the year or (ii) tilt angle adjusted monthly. Optimal angles for the PV arrays were obtained from the energy+ (https://energyplus.net/weather – Department of Energy, DoE) weather database. The effects of adding electronic equipment (*i.e.* a maximum power point tracker – MPPT- and DC/DC converter) were also evaluated. The simulation outcomes are reported in the next section.

Average solar-to-chemical efficiencies for all the considered cases [%]. Only non-zero values have been taken into account for the calculation. Standard deviation data are reported in Table S3 of the ESI

**Table:** 

	Fixed tilt			Monthly-adjusted tilt		
	4SHJ	5SHJ	MJ + Conc.	4SHJ	5SHJ	MJ + Conc.
Lausanne, CH	9.30	13.33	17.07	9.92	13.51	18.74
Phoenix, US	10.39	13.62	19.81	10.86	13.85	20.87
Delhi, IN	9.88	13.29	19.87	10.50	13.67	20.68

**Table:** 

	With MPPT and DC–DC converter					
Lausanne, CH	13.79	11.21	17.99	13.77	11.23	18.25
Phoenix, US	13.52	11.37	17.33	13.47	11.38	17.72
Delhi, IN	13.59	11.36	17.69	13.56	11.37	18.14

#### 3.2.1 Direct PV – electrolyzer coupling

PV cells were considered to directly drive electrochemical reactors in these simulations. No electronic power converters were used between the PVs and the electrolyzer.

SCEs are strongly dependent on the PV technology of choice. Yields as high as 10.5%, 15.1% and 24.9% were recorded for generators powered by 4SHJ, 5SHJ and MJ solar cells (Fig. S8 in the ESI†). These results were consistent with the experimental validations and literature. The efficiency differences are due to the PV intrinsic characteristics (*i.e.* the capability of delivering higher current densities) and the device configuration (*i.e.* relative position of working point and maximum power point). MJ-driven devices outperformed the considered alternatives. Additional details are reported in Fig. S10–S12 in the ESI.†

The simulated periodic adjustment of the PVs tilt angle throughout the year resulted in more robust and predictable operation. SCEs were roughly constant throughout the entire period (Fig. S9 in the ESI†). MJ-driven systems gained the most, as the optical concentrator could benefit from better incidence angles. Fig. S13–S15 in the ESI† report the detailed working current densities and SCEs.

Due to non-optimal working point (*V*_OP_ > *V*_MPP_), the 4SHJ-powered device could only provide a fraction of the hypochlorite generated by the 5SHJ and MJ-powered devices (65–70%, approximately); the series-connection of 4SHJ solar cells is therefore not appealing to drive an efficient solar-hypochlorite device.

The devices powered by MJ solar cells demonstrated the highest generation capabilities. Despite the performance degradation for non-normal incidence angles, the higher SCE yields ensured slightly superior hypochlorite throughput. The production discrepancy with 5SHJ-systems resulted to be negative in the considered temperate climate (Lausanne), due to larger angles of incidence (AOIs) for greater shares of the year and higher impact of the unexploited diffuse component on the global irradiance. The combination of the two effects equalized the chemical throughput of MJ-powered and 5SHJ-powered devices in such cases.

The external intervention necessary to adjust the PV tilt angle each month was beneficial for the chemical throughput as well (Table 2). Higher sodium hypochlorite productivities were recorded. The 4SHJ-powered devices were the most affected, as the sensitivity to even minimal fluctuations of *V*_OP_ was the highest. The NaClO production of MJ-driven system was relevantly boosted as well. The improvement relied on the better, on average, exposition of the solar concentrator. The 5SHJ-powered cases resulted to be the least-influenced; nevertheless, +3–6% gains were observed.

Sodium hypochlorite productivity for all the considered cases. The figures reported [g_NaClO_ per cm^2^ per year] are the yearly throughout obtained based on the energy + weather databases and are expressed in specific terms with respect to the PV input aperture (mini-module surface for 4SHJ and 5SHJ, solar concentrator input aperture area for MJ cells)

**Table:** 

	Fixed tilt			Monthly-adjusted tilt		
	4SHJ	5SHJ	MJ + Conc.	4SHJ	5SHJ	MJ + Conc.
Lausanne, CH	7.23	10.37	9.76	7.45	10.68	10.32
Phoenix, US	15.23	21.54	25.68	15.58	23.03	28.24
Delhi, IN	14.37	19.25	21.22	14.46	20.23	22.70

**Table:** 

	With MPPT and DC–DC converter					
Lausanne, CH	11.58	9.23	8.20	11.81	9.46	8.66
Phoenix, US	21.89	18.61	21.45	23.05	19.72	23.45
Delhi, IN	19.97	16.71	17.94	20.76	17.43	19.10

#### 3.2.2 PV – electrolyzer operation with MPPT and DC–DC converter

The influence of power electronics was simulated in the computational analysis. The addition of an MPPT coupled with a DC/DC converter can affect SCEs and the sodium hypochlorite generation. The introduction of electronic equipment can guarantee stable operation at high efficiencies; decoupling the generation and utilization ensures greater freedom with respect to the atmospheric conditions. However, the electronics introduce additional capital costs and degrade the SCE through the effect of the MPPT and DC/DC converter efficiencies (*η*_MPPT_ and *η*_DC–DC_, respectively). For the purpose of our study, we simulated an MPPT with 97% efficiency^40,41^ and a DC/DC converter with 90% efficiency;^42,43^ for the latter, we considered a switching converter and an average value of the current-dependent efficiency.

4SHJ-powered systems benefitted the most from power electronics; the working points could be externally shifted towards higher current (*i.e.* higher efficiencies) regimes. Improvements up to 150% and 160% were recorded for the average SCE yields and hypochlorite productivity, respectively (Fig. S16 in the ESI†).

Performance losses were also observed for 5SHJ and MJ-powered devices when power electronics was simulated. Peak efficiency values were decreased as a joint effect of *η*_MPPT_ and *η*_DC–DC_, to 12.4% and 21.0% for 5SHJ and MJ-powered devices, respectively (Fig. S18–S20 in the ESI†). In terms of yearly hypochlorite productivity, a 12.5–16.5% decrease was observed for 5SHJ and MJ-driven cases. This relied on the power loss due to the MPPT and DC/DC conversion losses (*η*_MPPT_*η*_DC–DC_, 15%, roughly). Based on our climate-based computations, we could assess that those devices performed better in direct coupling conditions, without electronic equipment to mediate the PV output.

The simulation of a monthly tracking strategy proved to be beneficial. It allowed an increase in the yearly average SCE by up to 2.5% and the NaClO productivity up to 9.3% (Fig. S17 and S21–S23 in the ESI†).

### 3.3 Upscaling and potential applications

The considered PV options can be upscaled to meet the demands of industrial electrochemical reactors. The technology of Si-based hetero-junction photovoltaics has considerably progressed in recent years and are foreseen to soon become a significant market player.^19,44^ Solar cells employing multi-junction technology have been object of extensive research and could become commercially available at scale in the near future.^45^ Furthermore, the optical concentrator we employed is particularly suitable for large-scale implementations due to its modularity.

We have evaluated the potential PV surface required to satisfy the water treatment needs of a small size hospital (40 patient beds) in two distinct locations; (i) a low-income country (*e.g.* India) and (ii) a developed country (*e.g.* Switzerland or the United States). For the first case we considered a 8000 liters daily water need (2.92 × 10^6^ liters per year), whereas for the developed countries we accounted for a requirement of 118.5 gallons per bed per year (17.92 × 10^6^ liters per year).^46^ We considered the addition of a sodium hypochlorite quantity of 50 mg L^−1^. It is worth noting that those dosages are much higher than those typically employed in water disinfection practices, but they are commonly considered for hospitals and health care facilities due to the fact that water is employed for cleaning and other utilizations, beside drinking.^47^

Table 3 reports the outcomes of the simulations. When PVs directly fed the electrochemical cell, 4SHJ-powered systems proved to be infeasible, as they require a much greater surface to obtain the targeted NaClO yearly production. Devices employing 5SHJ and MJ solar cells could meet the generation needs with surfaces of 8–9 m^2^ in Lausanne, 3–4 m^2^ in Phoenix and < 1 m^2^ in Delhi, depending on the required treatment severity.

PV surface [m^2^] required for a small size hospital (40 beds) in different locations. The calculated area is referred to the module effective surface for SHJ and to the solar concentrator input aperture for MJ solar cells. The two extremes of the reported interval refer to the least and most severe case scenario, respectively

**Table:** 

	Fixed tilt			Monthly-adjusted tilt		
	4SHJ	5SHJ	MJ + Conc.	4SHJ	5SHJ	MJ + Conc.
Lausanne, CH	12.4	8.6	9.2	12.0	8.4	8.7
Phoenix, US	5.9	4.2	3.5	5.8	3.9	3.2
Delhi, IN	1.0	0.8	0.7	1.0	0.7	0.6

**Table:** 

	With MPPT and DC–DC converter					
Lausanne, CH	7.7	9.7	10.9	7.6	9.5	10.3
Phoenix, US	4.1	4.8	4.2	3.9	4.5	3.8
Delhi, IN	0.7	0.9	0.8	0.7	0.8	0.8

The addition of power electronics to drive the operation significantly restrained the required PV surface for 4SHJ-powered devices. In this configuration, due to high current density throughput and the absence of angular limitations, they could outperform the competitive PV technologies.

The parallel-connection of several 4SHJ, 5SHJ or MJ solar modules was considered in the assessments mentioned above. This would allow the system to maintain identical *V*_OC_ values and increase the output current, but it poses issues regarding the current density circulating into the module busbars. Considering an ideal brine electrolyzer (Tafel slope ∼ 30 mV (ref. 47)), we could conclude that maintaining identical *V*_OC_s is thermodynamically feasible even at high amperage. Taking into account the same module area than commercial products (1.5 m^2^), the module busbars would need to be sized accordingly; this results in a cross-section 15–40 times larger than that employed in high-efficiency, commercially available c-Si modules (a maximum current density of 5 A mm^−2^ was considered, referred to the busbar cross section area).^48^ In case the deployment of identical busbars is preferable to using commercial modules, the surface limit would be approx. 500 cm^2^, 800 cm^2^ and 380 cm^2^ for 4SHJ, 5SHJ and MJ, respectively. High amperages and limited *V*_OC_s are desirable to guarantee high conversion yields without power electronics; these constraints become practically feasible once the solar module's electrical architecture has been correctly dimensioned.

### 3.4 Technoeconomic analysis of solar-powered hypochlorite devices

The economic viability of solar-powered devices for sodium hypochlorite production was assessed. We aimed at evaluating the levelized production cost of a 12% hypochlorite solution (LC_NaClO,12%_) and the specific cost of water disinfection. The test case scenario of choice was a small size hospital, similarly to the previous section.

The technoeconomic evaluation considered the cost of semiconductors, materials, labor, electronics, as well as the balance of system (BoS). The expenses associated with solar tracking, solar concentrator and additional control equipment were included for the devices powered by MJ cells. The sodium hypochlorite productivities and required PV areas presented in Tables 2 and 3, respectively, were taken into account.

Calculations were based on the net present value (NPV); the LC_NaClO,12%_ was calculated adjusting the value such that the NPV of capital, operating expenses (OCi, at year i) and revenues (PRi, at year i) summed to zero.7
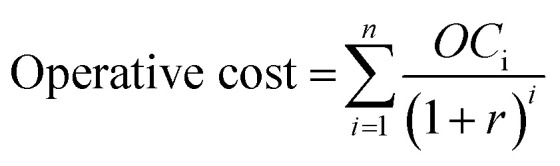
8
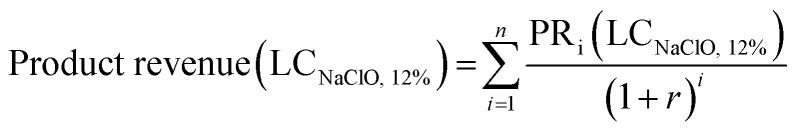

9 NPV = Product revenue − operative cost − capital = 0

A discount factor, *r* = 1.9%, and a 20 year device lifetime were considered.^2^ The operative costs included standard operation and maintenance (O&M) of sodium hypochlorite plants,^49^ as well as a mid-lifetime catalyst and DC–DC converter (if present) replacement. This assumption was consistent with the average DSA lifespan^50^ and the warranty period of electronics suppliers. The technoeconomic analysis details are provided in Tables S4 and S5 of the ESI.†

The production costs ($ ton^−1^) of a 12% sodium hypochlorite solution generated using solar power are reported in Table S6 of the ESI.† The benchmark for comparison was the generation cost of a similar hypochlorite solution *via* electrochemical routes at the industrial scale, which was estimated to be $ 195 ton^−1^_NaClO_.^51,52^

The solar-powered production of NaClO in a moderate irradiance climate (*i.e.* Lausanne) proved to be economically penalized by lower solar irradiance and could never outcompete the benchmark value.

In higher irradiance climates (*i.e.* Phoenix and Delhi), solar-driven devices powered by 5SHJ or MJ modules were cost-competitive solutions, especially in the case of direct coupling between PV arrays and electrolyzers. Production costs were as low as $ 129 ton^−1^_NaClO_, thus more advantageous than traditional electrochemical routes powered by grid electricity.

The introduction of power electronics resulted in higher average costs, due to increased capital, O&M expenses and associated electronics inefficiencies. Modules with 4SHJ in series were the most viable PV technology in such cases; production costs were within $ 157–197 ton^−1^_NaClO_.

Similar considerations apply for the cost of water disinfection ($ m^−3^_water_). It was evaluated assuming a hypochlorite dosage of 50 mg L^−1^. Results are reported in Table S7 of the ESI.† Solar-driven water disinfection costs were as low as c$ 5.4 m^−3^_water_. Those values were consistent with those of previous literature.^53^

### 3.5 Cost comparison between chlorination and ultraviolet radiation for water disinfection

Ultraviolet (UV) technologies for water disinfection have emerged in recent years, due to important leaps in lamp performance and reliability.^54–56^ UV radiation is now commonly the second most utilized technology for water disinfection, after chlorination.^49,53^

The economic viability of a solar-powered UV disinfection device was assessed and compared with the outcomes presented in the previous section. We took into account the case of a small size hospital (identical to the previous sections) for our simulations.

Three different UV lamp technologies were considered, based on the pressure inside the lamp and the light emission intensity: low pressure–low intensity (LP–LI), low pressure–high intensity (LP–HI) and medium pressure–high intensity (MP–HI). Low pressure lamps generally have lengths of 0.75–1.5 m, with diameters of 1.5–2.0 cm; high pressure lamps are more compact and can emit up to 15 times the intensity of low pressure systems. The emission peak is at *λ* = 253.7 nm for all technologies.

The technoeconomic inputs of the UV chamber are reported in Table S8 of the ESI.† The required PV area for each technology and location, and the considered number of lamps are presented in Tables S9 and S10 of the ESI,† respectively.

The cost of solar-powered UV water disinfection ($ m^−3^_water_) was assessed for comparison with solar-driven chlorination; a device lifetime of 20 years and a discount rate, *r* = 1.9%, were considered. Only applications with power electronics were took into account, as UV lamps require high voltage (240–480 volts) to operate. Direct PV coupling was therefore impossible. Outcomes are reported in Table S11 of the ESI.†

Solar-UV devices were demonstrated to provide more expensive water disinfection means than solar-driven chlorination. Depending on the technology of choice, costs were as low as c$11 m^−3^_water_. This was consistent with similar studies reported in literature.^57–59^ Solar-electrochemical routes to generate hypochlorite species with the purpose of water disinfection are therefore more economically viable and advantageous for such on-site applications. Along with the economic advantage, deployment of solar-powered UV devices for off-grid applications in low-income countries could be hampered by (i) the lack of any residual disinfectant into the water and (ii) the greater need of components supply and maintenance. The first is particularly essential in low-income countries, because water is not often sanitized at the point of use but, more commonly, in a centralized station and then distributed. Since water safety can be compromised during transportation, a residual disinfectant is required to prevent downstream contamination. Chlorinated compounds can provide the residual disinfection effect if properly dosed. Transporting materials and parts to isolated locations also appear to be a severe bottleneck for the diffusion of solar-UV technologies in third world countries.

## 4. Conclusions

This study investigated the technical and economical operation of solar-powered sodium hypochlorite generators employing Si-based or GaAs-based PV technologies. The highest SCE ratios were obtained for MJ-driven devices, outperforming 4SHJ and 5SHJ-powered systems.

4SHJ modules proved to be impractical to directly power a solar-hypochlorite device, as they operate at *V*_OP_ > *V*_MPP_, resulting in poor efficiency and limited product throughput. In terms of chemical productivity, the gap between 5SHJ-powered and MJ-powered generators was considerably lower than the SCE difference. This was due to the optical acceptance of the solar concentrator employed for GaAs-based PVs, as well as to the diffuse component of solar irradiance that is exploited in Si cells, but could not be captured by the concentrator optics. Correcting the tilt of solar modules throughout the year (*e.g.* monthly) resulted in relevant benefits; the strategy could boost the average SCEs and hypochlorite production up to 10%.

The deployment of power electronics (MPPT and DC/DC converter) to assist the operation benefitted 4SHJ-powered devices the most; this resulted in increased average SCEs and NaClO productivity throughout the year. The operation of 5SHJ-powered and MJ-powered generators proved to be feasible in conjunction with power electronics; nevertheless, the operation suffered from the energy losses in the voltage conversion process, and failed to reach the outcomes of the direct coupling case. This demonstrated that thorough components design targeting load-matching could bypass the implementation of additional electronics and result in higher chemical throughput.

The possibility of implementing solar-powered sodium hypochlorite generators in real applications was also evaluated. For the test case, we considered a small-scale hospital (40 patients), in both a developed and low-income country. The results highlighted that in direct coupling operation, 5SHJ and MJ solar cells were the preferred PV technologies of-choice, as they could restrain the required PV surface. Only in cases where electronics were simulated to mediate the output were 4SHJ-powered devices more desirable than the alternatives.

The technoeconomic analysis highlighted that sodium hypochlorite could be generated at a competitive cost *via* solar-driven electrochemical routes, when compared to hypochlorite generated electrochemically at the industrial scale. Additionally, the technology proved to outcompete the benchmark of solar-powered UV disinfection. Simple devices, maintenance, and the number of components are likely to represent other key decision factors in off-grid, independent installations.

This comparative analysis highlighted the technical benefits of solar-powered operation for stand-alone sodium hypochlorite generators. Due to their low cost and reliability, high-efficiency SHJ technologies are the current best candidates to have an impact in the near-term, with GaAs-powered installations likely to follow similar pathways once their commercial potential starts to be fully exploited.

The series-connection of five hetero-junction cells proved to be the best technological option in direct operation in a short-term implementation scenario; when electronics were employed in the system, the mini-module with four SHJ in series represented the best alternative. The benefits introduced by MPPT's and DC/DC converters are nevertheless associated with significant capital expenses, due to higher initial investments and operation costs. The addition of electronic regulators is therefore undesirable. Regardless of the technology of choice, thorough component sizing and matching can circumvent the implementation of electronics and is essential to guarantee working potentials lower than *V*_MPP_. This condition ensures stability of operation and limited efficiency fluctuations.

Independent, stand-alone, off-grid systems such as those demonstrated in the study can have a broad and immediate impact on communities where treatment of water effluents carrying waterborne diseases is necessary, but grid electricity is remote, or bulk hypochlorite supplies are inaccessible. The proposed technology could affect communities whose access to potable drinking water is hampered by the contamination of organic pathogens. The work follows the guidelines dictated by the United Nations Sustainable Development Goals.

Centralized facilities would also benefit from PV technologies penetration in the chemical and electrochemical industry, as they could operate by exploiting cheap solar electricity when the source is available. Nevertheless, grid-backup and/or batteries would be required to fulfill appropriate capacity factors and guarantee continuous operability.

The solar-driven devices we have demonstrated experimentally and assessed computationally represent a new technological platform that can pave the way towards autonomous hypochlorite generators for removal of waterborne organic pathogens, and spur further implementation of renewable energy sources in the electrochemical industry.

## Conflicts of interest

The authors declare that three co-authors (L. Coulot, M. Ackermann, F. Gerlich) are the founders of a private solar concentration company (Insolight SA). We declare that their contribution was limited to the provision and the operation of the multi-junction solar cells, and they also provided support in analyzing the meteorological data. Nevertheless, they were not involved in the manuscript writing, in order to avoid any unintended bias.

## Supplementary Material

RA-009-C9RA02221J-s001
