# Supplementary material for: A comparative performance analysis of stand-alone, off-grid solar-powered sodium hypochlorite generators
Source: RSC Adv. 2019 May 8;9(25):14432–42. doi: 10.1039/c9ra02221j (PMC9064146; doi:10.1039/c9ra02221j)
Supplement: RA-009-C9RA02221J-s001 [file RA-009-C9RA02221J-s001.pdf]

Electronic Supplementary Material (ESI) for RSC Advances.
This journal is © The Royal Society of Chemistry 2019

Supplementary Material for

A comparative performance analysis of stand-alone, off-grid solar-powered sodium hypochlorite generators

E. Chinello,[a] M. A. Modestino,[b] J.W. Schüttauf,[c] L. Coulot,[d] M. Ackermann,[d] F. Gerlich,[d] A. Faes,[c] D. Psaltis[a] and C. Moser[a]

a. *School of Engineering, École Polytechnique Fédérale de Lausanne (EPFL), Station 17, 1015, Lausanne, Switzerland. Email: enrico.chinello@epfl.ch. Tel: +41 21 69 35171.*

b. *Tandon School of Engineering, New York University (NYU); Rogers Halls 600A, Brooklyn, 11201 NY.*

c. *Swiss Center for Electronics and Microtechnology (CSEM), Rue Jaquet-Droz 1, CH 2002 Neûchatel, Switzerland.*

d. *Insolight SA, Chemin de la Raye 13, Ecublens (VD), CH 1015 Lausanne, Switzerland.*

The document contains Table S1-S11 and Figures S1 – S30.

Table S1: List of abbreviations

List of abbreviations

PV	Photovoltaic
DSA	Dimensionally Stable Anode
c-Si	Crystalline Silicon
SCE	Solar-to-chemical conversion efficiency
V_{OC}	Open circuit voltage
SHJ	Silicon hetero-junction (solar cells)
MJ	Multi-junction (solar cells)
$\eta_{Faraday}$	Faradaic efficiency (or Current efficiency)
GHI	Global Horizontal Irradiance
DHI	Direct horizontal irradiance
DNI	Direct Normal Irradiance
$\eta_{optical}$	Optical efficiency (of optical elements)
VOP	Operative (or working) voltage
MPP	Maximum power point
VMPP	Maximum power point voltage
AOI	Angle of Incidence
MPPT	Maximum power point tracker
η_{MPPT}	Maximum power point tracker efficiency
$\eta_{DC\text{-}DC}$	DC-DC converter efficiency
$LC_{NAClO,12\%}$	Levelized cost of a 12% sodium hypochlorite solution
BoS	Balance of system
NPV	Net present value
O&M	Operation and maintenance
UV	Ultraviolet
LP-LI	Low pressure - low intensity (UV technology)
LP-HI	Low pressure - high intensity (UV technology)
MP-HI	Medium pressure - high intensity (UV technology)

Table S2: PV array tilt throughout the year in case the tilt is adjusted monthly. All angles are measured with respect to the horizontal plane.

	**Lausanne**	**Phoenix**	**Delhi**
**January**	62°	49°	45°
**February**	54°	41°	37°
**March**	46°	33°	29°
**April**	38°	25°	21°
**May**	30°	17°	13°
**June**	22°	10°	6°
**July**	30°	17°	13°
**August**	38°	25°	21°
**September**	46°	33°	29°
**October**	54°	41°	37°
**November**	62°	49°	45°
**December**	70°	56°	52°

Table S3: Standard deviations for the average solar-to-chemical efficiencies (SCE) reported in Table 2 in the main manuscript [%].

	Fixed Tilt			*Monthly-adjusted Tilt*		
	4SHJ	*5SHJ*	*MJ + Conc.*	*4SHJ*	*5SHJ*	*MJ + Conc.*
Lausanne, CH	2.72	4.38	6.43	2.72	4.40	6.36
Phoenix, US	2.70	3.18	5.51	2.80	3.36	5.81
Delhi, IN	2.72	3.58	6.04	2.91	3.62	6.02
	With MPPT and DC-DC converter					
Lausanne, CH	0.41	0.38	1.79	0.41	0.37	1.88
Phoenix, US	0.43	0.31	2.11	0.44	0.30	2.07
Delhi, IN	0.41	0.30	1.81	0.43	0.29	1.77

Table S4: Technoeconomic analysis input parameters for devices employing SHJ solar cells.

SHJ - Powered Devices

Photovoltaics		
PV Modules[1]	96	$\$\cdot m^{-2}$
Wiring[2,3]	16	$\$\cdot m^{-2}$
Panel Mounting Materials[2,3]	29	$\$\cdot m^{-2}$
PV Labor Installation[2,3]	29	$\$\cdot m^{-2}$
Other PV soft BoS[2,3]	56	$\$\cdot m^{-2}$
Electronics (if present)		
DC-DC Converter + MPPT[4]	51	$\$\cdot m^{-2}$
Electrochemical reactor		
Chassis[5]	6.6	$\$\cdot m^{-2}$
Catalysts (DSA/DSA)	125	$\$\cdot m^{-2}$
Electrolyzer Hard BoS[4]	61	$\$\cdot m^{-2}$
Electrolyzer Installation Labor[4]	19	$\$\cdot m^{-2}$

All costs are referred to the photovoltaic module area. Catalysts (dimensionally stable anode – DSA) costs were derived from quotes of industrial suppliers.

Table S5: Technoeconomic analysis input parameters for devices employing MJ solar cells.

MJ - Powered Devices

Photovoltaics		
Window (antireflective glass)[6]	5	$\$\cdot m^{-2}$
III-V cells[7]	175	$\$\cdot m^{-2}$
Tracker – Hardware[3]	44.8	$\$\cdot m^{-2}$
Concentrator[8]	48	$\$\cdot m^{-2}$
Control Systems[9]	8.9	$\$\cdot m^{-2}$
Wiring[2,3]	16	$\$\cdot m^{-2}$
Panel Mounting Materials[2,3]	29	$\$\cdot m^{-2}$
PV Labor Installation[2,3]	29	$\$\cdot m^{-2}$
Other PV soft BoS[2,3]	56	$\$\cdot m^{-2}$
Electronics (if present)		
DC-DC Converter + MPPT[4]	51	$\$\cdot m^{-2}$
Electrochemical reactor		
Chassis[5]	6.6	$\$\cdot m^{-2}$
Catalysts (DSA/DSA)	125	$\$\cdot m^{-2}$
Electrolyzer Hard BoS[4]	61	$\$\cdot m^{-2}$
Electrolyzer Installation Labor[4]	19	$\$\cdot m^{-2}$

All costs are referred to the photovoltaic module area. Catalysts (dimensionally stable anode – DSA) costs were derived from quotes of industrial suppliers.

Table S6: Solar-powered production cost on a 12% sodium hypochlorite solution. All costs are $·ton^{-1}.

	Fixed Tilt			*Monthly-adjusted Tilt*		
	4SHJ	*5SHJ*	*MJ + Conc.*	*4SHJ*	*5SHJ*	*MJ + Conc.*
Lausanne, CH	499	346	370	483	338	350
Phoenix, US	237	169	141	233	157	129
Delhi, IN	247	173	165	233	170	159
	With MPPT and DC-DC converter					
Lausanne, CH	310	390	438	306	382	414
Phoenix, US	165	193	169	157	181	153
Delhi, IN	197	200	192	178	185	185

Table S7: Water disinfection cost using a solar-generated 12% sodium hypochlorite solution. All costs are $c\$ \cdot m_{water}^{-3}$.

	Fixed Tilt			*Monthly-adjusted Tilt*		
	4SHJ	*5SHJ*	*MJ + Conc.*	*4SHJ*	*5SHJ*	*MJ + Conc.*
Lausanne, CH	20.8	14.4	15.4	20.1	14.1	14.6
Phoenix, US	9.9	7.0	5.9	9.7	6.5	5.4
Delhi, IN	10.3	7.2	7.1	9.7	7.1	6.6
	With MPPT and DC-DC converter					
Lausanne, CH	12.9	16.3	18.3	12.8	15.9	17.3
Phoenix, US	6.9	8.0	7.0	6.5	7.5	6.4
Delhi, IN	8.2	8.3	8.2	7.4	8.2	7.6

Table S8: Technoeconomic inputs for the solar-powered UV disinfection devices.

SHJ - Powered Devices

**Photovoltaics**			
PV Modules[1]	96	$\$\cdot m^{-2}$	
Wiring[2,3]	16	$\$\cdot m^{-2}$	
Panel Mounting Materials[2,3]	29	$\$\cdot m^{-2}$	
PV Labor Installation[2,3]	29	$\$\cdot m^{-2}$	
Other PV soft BoS[2,3]	56	$\$\cdot m^{-2}$	
Electronics (if present)			
DC-DC Converter + MPPT[4]	51	$\$\cdot m^{-2}$	
**UV chamber**			
	LP-LI	***LP-HI***	***MP-HI***
Cost per lamp [$]	175	200	250
Construction Cost [% of lamps cost]	150%	150%	150%
**O&M UV chamber (each year)**			
Replacements			
Lamps [% of lamps cost]	25%	25%	25%
Ballast [% of lamps cost]	5%	5%	5%
Sleeve [% of lamps cost]	5%	5%	5%
Other			
Chemicals [$]	25	25	25
Labor [% of lamps cost]	15%	15%	15%
Misc. Repair [% of lamps cost]	5%	5%	5%

MJ - Powered Devices

**Photovoltaics**			
Window (antireflective glass)[6]	5	$\$\cdot m^{-2}$	
III-V cells[7]	175	$\$\cdot m^{-2}$	
Tracker – Hardware[3]	44.8	$\$\cdot m^{-2}$	
Concentrator[8]	48	$\$\cdot m^{-2}$	
Control Systems[9]	8.9	$\$\cdot m^{-2}$	
Wiring[2,3]	16	$\$\cdot m^{-2}$	
Panel Mounting Materials[2,3]	29	$\$\cdot m^{-2}$	
PV Labor Installation[2,3]	29	$\$\cdot m^{-2}$	
Other PV soft BoS[2,3]	56	$\$\cdot m^{-2}$	
Electronics (if present)			
DC-DC Converter + MPPT[4]	51	$\$\cdot m^{-2}$	
**UV chamber**			
	LP-LI	***LP-HI***	***MP-HI***
Cost per lamp [$]	175	235	270
Construction Cost [% of lamps cost]	150%	150%	150%
**O&M UV chamber (each year)**			
Replacements			
Lamps [% of lamps cost]	25%	25%	25%
Ballast [% of lamps cost]	5%	5%	5%
Sleeve [% of lamps cost]	5%	5%	5%
Other			
Chemicals [$]	25	25	25
Labor [% of lamps cost]	15%	15%	15%
Misc. Repair [% of lamps cost]	5%	5%	5%

Data for the capital expense and O&M of the UV chamber were adapted and derived from literature[10,11], taking into account the considered operative flow rate.

1 Price Quotes_EnergyTrend PV, https://pv.energytrend.com/pricequotes.html, (accessed February 8, 2018).
2 R. Margolis, D. Feldman and D. Boff, *Q4 2016/Q1 2017 Solar Industry Update*, National Renewable Energy Laboratory - NREL, 2017.
3 Solar Market Insight Report 2014 Q2, /research-resources/solar-market-insight-report-2014-q2, (accessed September 22, 2017).
4 W. G. Colella and J. M. Moton, .

5 Injection Molded Plastic Market | Industry Report, 2022, http://www.grandviewresearch.com/industry-analysis/injection-molded-plastics-market, (accessed September 22, 2017).
6 Solar Glass Price Plunge to Cease as Trade Sanctions Take Effect | IHS Online Newsroom, http://news.ihsmarkit.com/press-release/design-supply-chain-media/solar-glass-price-plunge-cease-trade-sanctions-take-effect, (accessed September 22, 2017).
7 M. Woodhouse and A. Goodrich, *A Manufacturing Cost Analysis Relevant to Single- and Dual-Junction Photovoltaic Cells Fabricated with III-Vs and III-Vs Grown on Czochralski Silicon*, The Renewable Energy Laboratory (NREL), U.S.A., 2013.
8 C. Turchi, *Parabolic Trough Reference Plant for Cost Modeling with the Solar Advisor Model (SAM)*, The Renewable Energy Laboratory (NREL), U.S.A., 2010.
9 B. D. James, G. N. Baum, J. Perez and K. N. Baum, *DOE Rep.*
10 C. Solomon, P. Casey, C. Mackne and A. Lake, *Ultraviolet disinfection*, .
11 *Evaluation of Ultraviolet (UV) radiation disinfection Technologies for Wastewater Treatment Plant Effluent*, New York State Energy Research and Development Authority (NYSERDA), New York, 2004.

Table S9: Photovoltaic surface required to power UV disinfection devices. All data are m^2.

	Low pressure - Low Intensity lamps					
	Fixed Tilt			*Monthly-adjusted Tilt*		
	4SHJ	*5SHJ*	*MJ + Conc.*	*4SHJ*	*5SHJ*	*MJ + Conc.*
Lausanne, CH	2.74	3.41	3.84	2.69	3.34	3.63
Phoenix, US	1.48	1.77	1.46	1.4	1.66	1.33
Delhi, IN	0.33	0.39	0.35	0.32	0.38	0.33
	Low pressure - High intensity lamps					
Lausanne, CH	2.35	2.93	3.29	2.31	2.86	3.11
Phoenix, US	1.27	1.52	1.25	1.2	1.42	1.14
Delhi, IN	0.28	0.34	0.3	0.27	0.32	0.28
	Medium pressure - High intensity lamps					
Lausanne, CH	8.61	10.73	12.07	8.45	10.49	11.41
Phoenix, US	4.66	5.56	4.59	4.39	5.21	4.17
Delhi, IN	1.03	1.24	1.11	0.99	1.18	1.04

Table S10: Number or lamps required for each technology and location. Data were extrapolated for the design flow rate from literature[11].

	Low pressure - Low Intensity	*Low pressure – High Intensity*	*Medium pressure – High Intensity*
Lausanne, CH	16	10	6
Phoenix, US	16	10	6
Delhi, IN	4	3	3

Table S11: Cost of water disinfection using solar-powered UV radiation. All values are $\$ \cdot m_{water}^{-3}$.

	Low Pressure – Low Intensity (LP-LI) UV Lamps					
	Fixed Tilt			*Monthly Adjusted tilt*		
	4 SHJ	*5 SHJ*	*MJ + Conc.*	*4 SHJ*	*5 SHJ*	*MJ + Conc.*
Lausanne, CH	17.7	17.8	17.9	14.9	14.9	15.1
Phoenix, US	17.5	17.6	17.6	14.7	14.8	14.8
Delhi, IN	22.5	22.6	22.6	22.5	22.6	22.6
	Low Pressure - High-Intensity (LP-HI) UV Lamps					
Lausanne, CH	14.9	14.9	15.1	14.9	14.9	15.1
Phoenix, US	14.7	14.8	14.8	14.7	14.8	14.8
Delhi, IN	22.7	22.7	22.7	22.6	22.7	22.7
	Medium Pressure - High-Intensity (MP-HI) UV Lamps					
Lausanne, CH	11.1	11.4	12	11.1	11.4	11.9
Phoenix, US	10.7	10.8	10.8	10.6	10.7	10.7
Delhi, IN	26.3	26.4	26.6	26.3	26.4	26.5

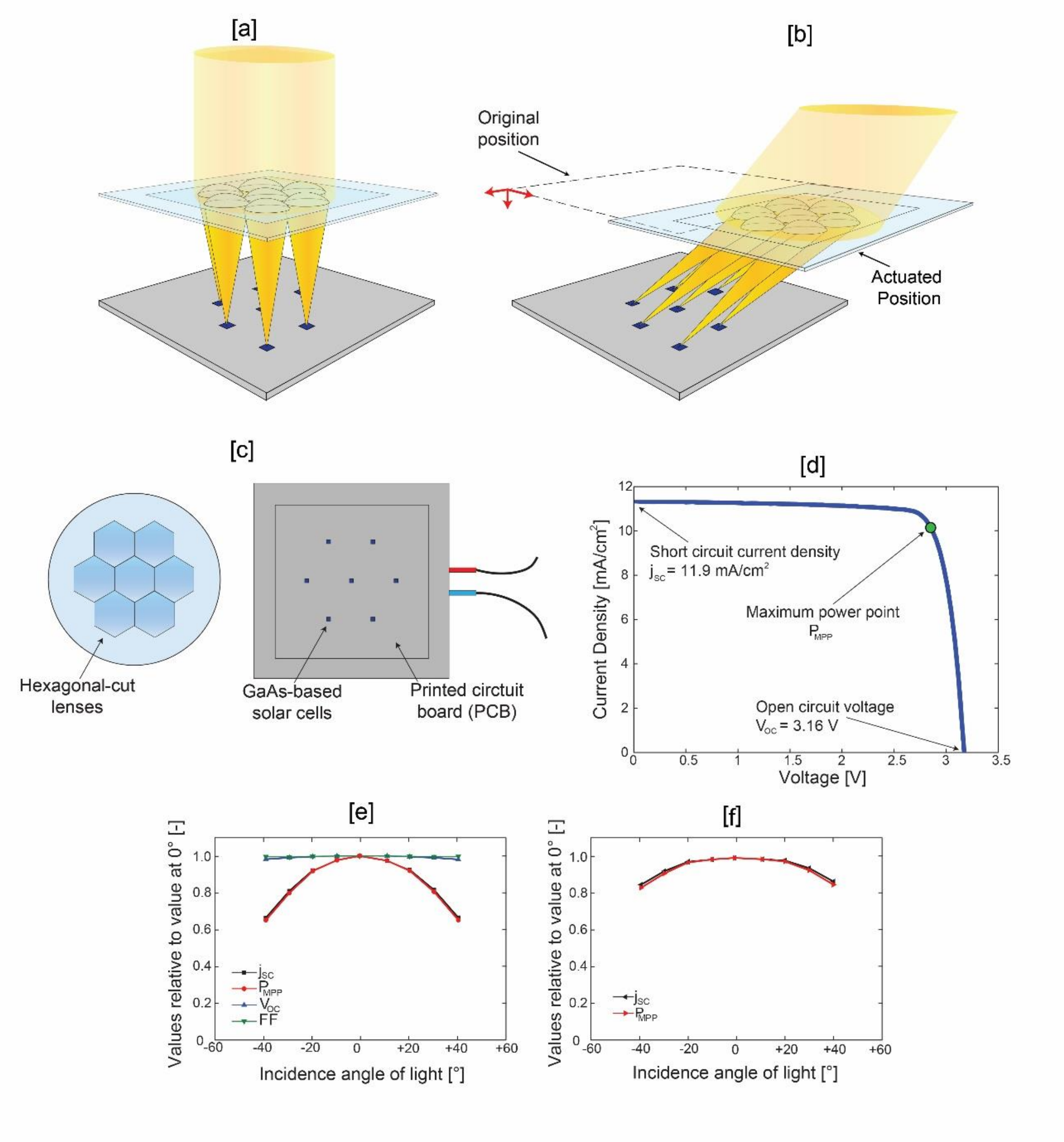

Figure S1: Insolight optical concentrator characteristics. [a] and [b] report the concentrator working principle, with the optics moving linearly going from normal incidence to tilted illumination. [c]: overview of employed prototype. [d]: electrical characteristics recorded and certified by Fraunhofer ISE (Freiburg, Germany). [e]: influence of incidence angle on short circuit current density (j_{SC}), power at maximum power point (P_{MPP}), open-circuit voltage (V_{OC}) and fill factor (FF). [f]: values in [e] corrected for the effective input aperture area. Data in [e] and [f] were certified by Fraunhofer ISE (Freiburg, Germany).

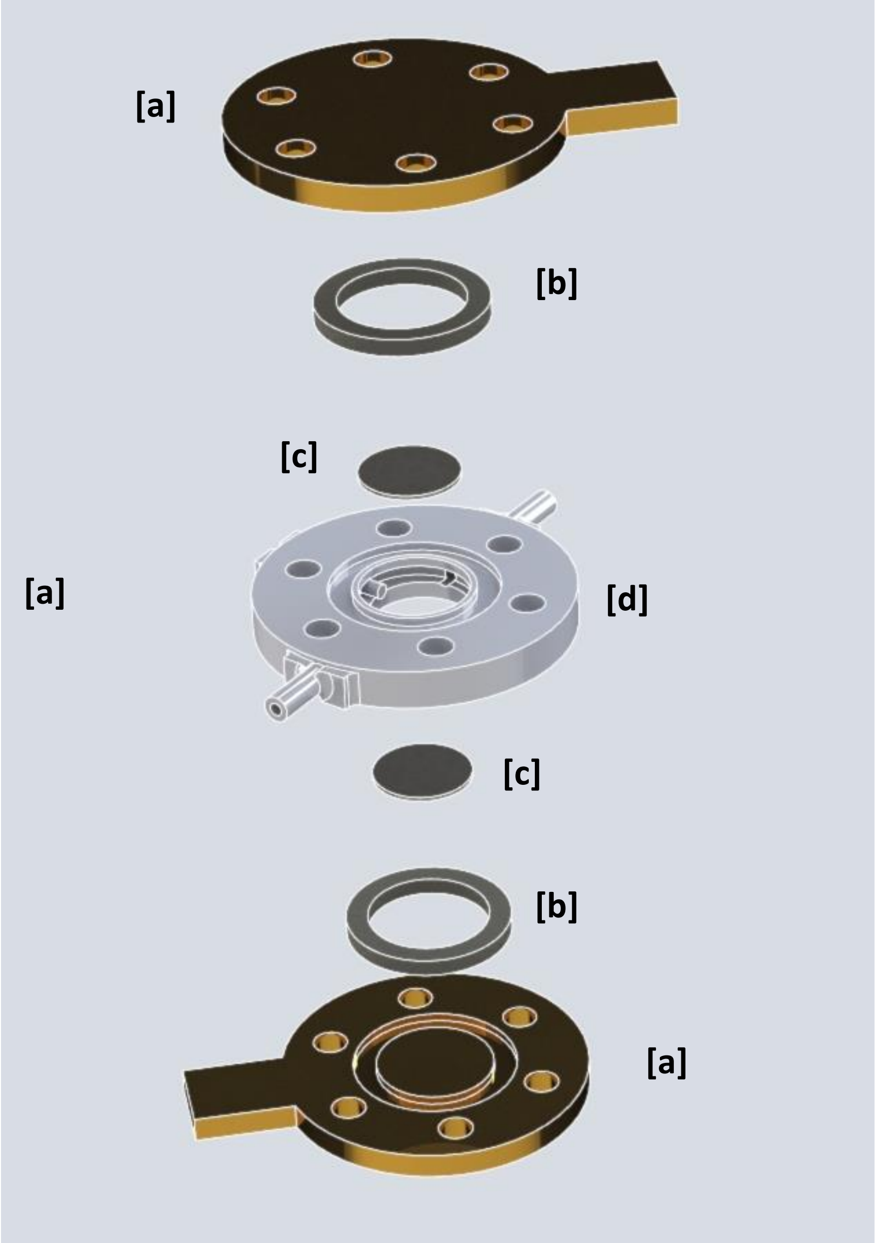

Figure S2: Schematic overview of the electrochemical cell for sodium hypochlorite generation. [a]: conductive plates (steel, electroplated in gold) to host the electrodes and conduct the charges. [b]: nitrile-rubber (NBR) O-rings. [c]: electrodes. [d]: separator, to hold the electrodes at the correct distance.

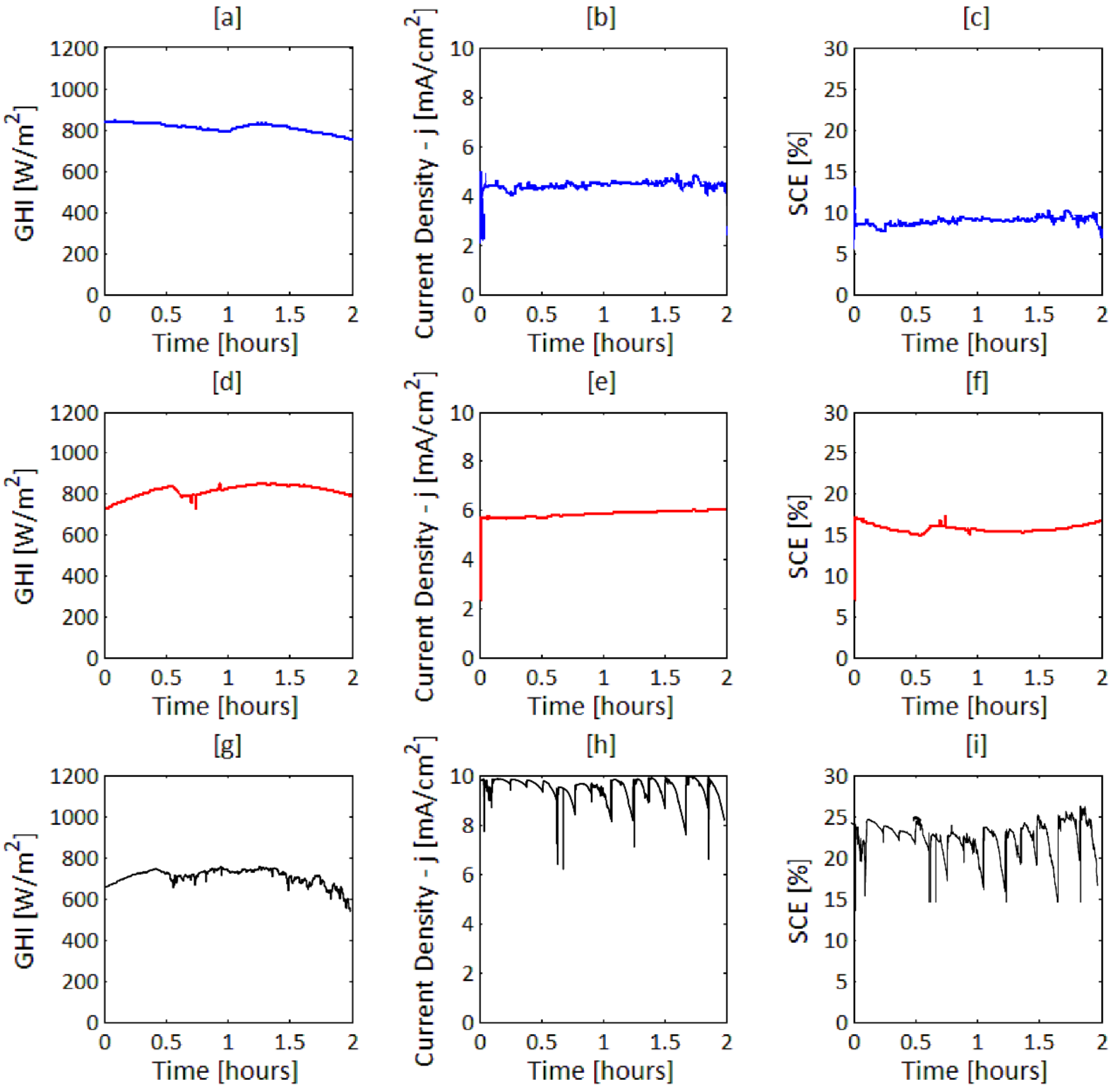

Figure S3: Solar irradiance, measured current density and measured solar-to-chemical efficiency for the experiments conducted on June 26th 2017 and July 5th 2017 under real atmospheric conditions. [a], [b], [c] report the global horizontal irradiance (GHI), working current density (j) and SCE for the 4SHJ-powered device, recorded on June 26th 2017, 1 – 3 PM. [d], [e], [f] report the global horizontal irradiance (GHI), working current density (j) and SCE for the 5SHJ-powered device, recorded on June 26th 2017, 11AM – 1 PM. [g], [h], [i] report the direct normal irradiance (DNI) corrected for the concentrator optical efficiency, working current density (j) and SCE for the MJ-powered device, recorded on July 5th 2017, 12-2 PM.

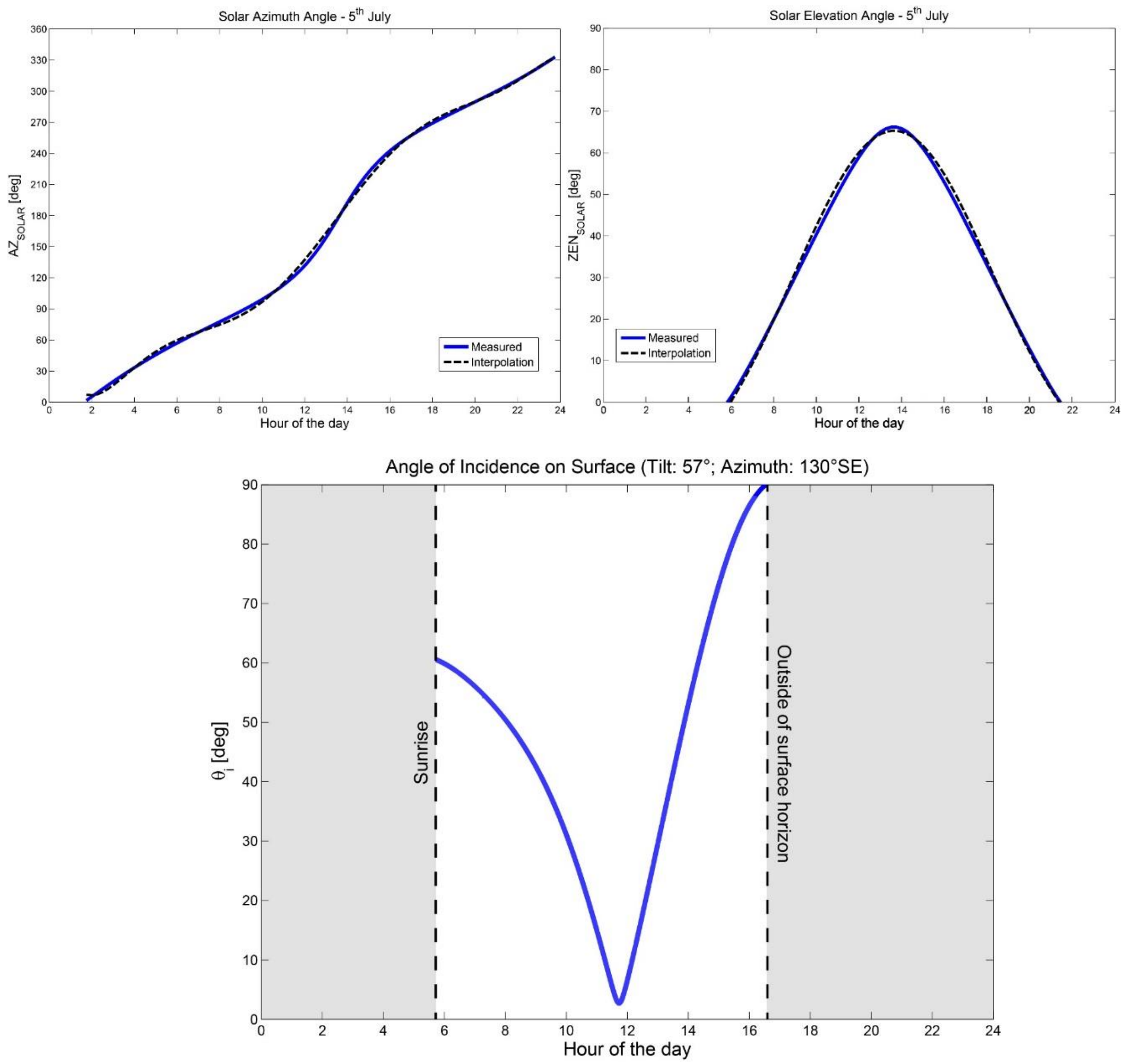

$$AOI = cos^{-1}[\cos(ZEN_{Solar}) \cdot \cos\left(Tilt_{array}\right) + \sin(ZEN_{Solar}) \cdot \sin(Tilt_{array}) \cdot \cos(AZ_{Solar-}AZ_{array})$$

Figure S4: Solar azimuth and elevation angle for July 5th 2017, in Lausanne (CH). Values have been utilized to compute the angle of incidence on the surface (AOI) according to the reported formula. The angle of incidence have been calculated to assess the optical efficiency of the solar concentrator.

Range (nm)	ASTM Standard (%)	Solar Simulator (%)
**400-500**	16.9 %	22.97 %
**500-600**	19.7 %	20.66 %
**600-700**	18.5 %	18.03 %
**700-800**	15.2 %	13.91 %
**800-900**	12.9 %	11.59 %
**900-1100**	16.8 %	12.83 %
**SUM**	100 %	100 %

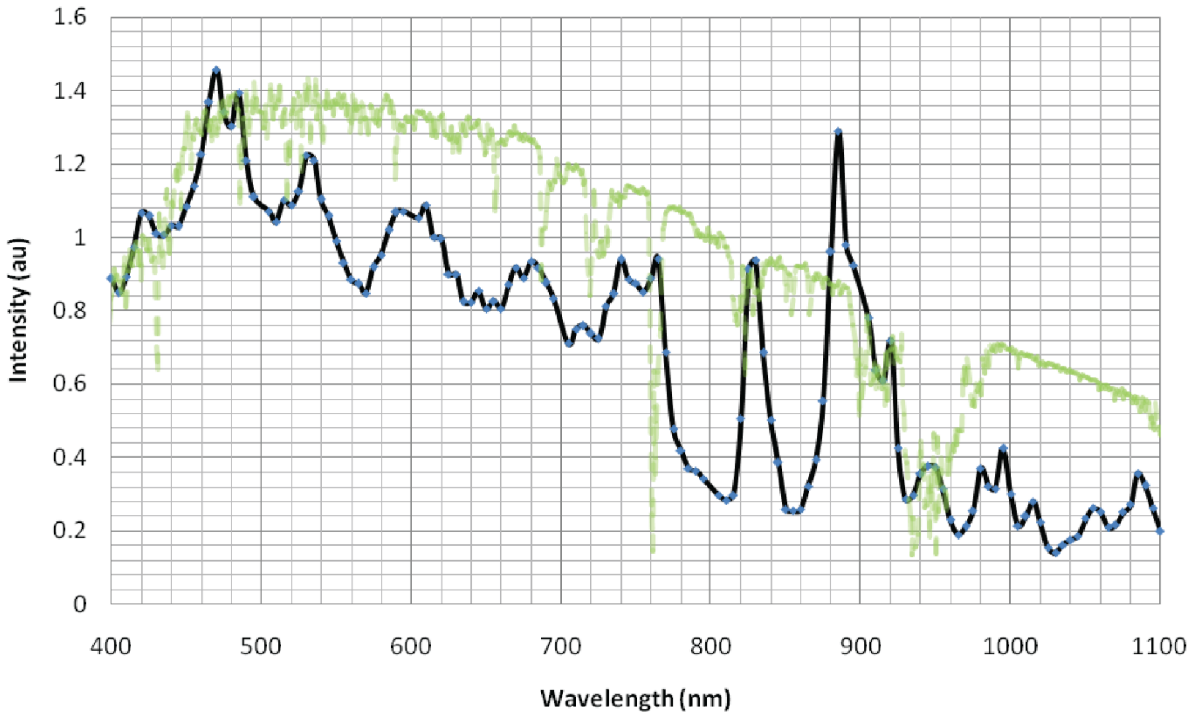

Figure S5: Sciencetech SF300 solar simulator spectral data. The ASTM standard for direct normal incidence dictates that for Class A spectral match to be achieved the percentage of power contained in each interval set out by the standard must deviate no more than ±25% from the standard. The results are the measured spectrum of our lens based Sciencetech SF300 solar simulator using a Xenon short-arc lamp. The results of the measurement indicate a Class A spectral match based on the ASTM standard for global irradiation. The provided graph shows the measured spectrum of the solar simulator (black) and the solar reference spectrum (green) for AM1.5G.

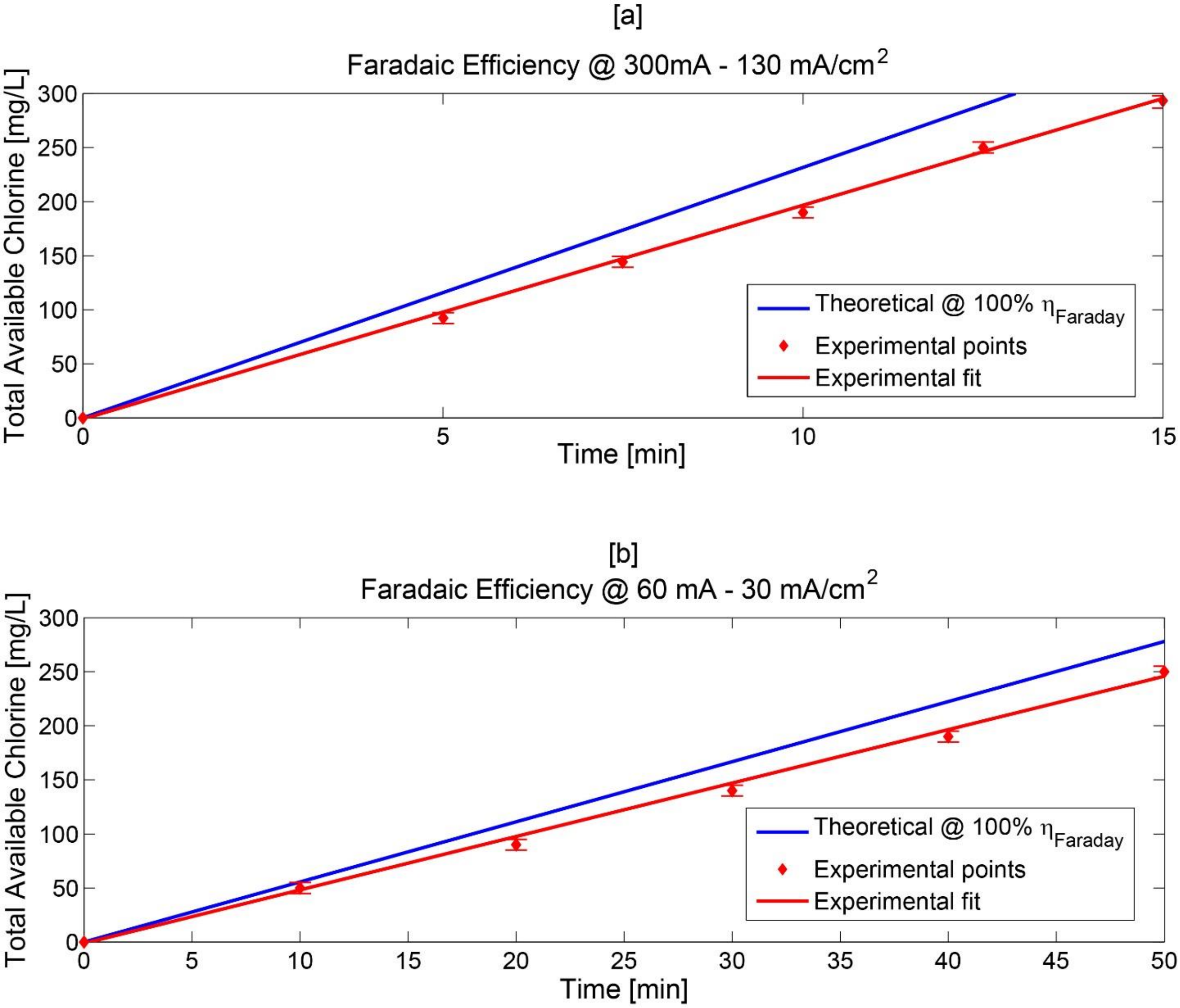

Figure S6: Faradaic efficiencies of solar-hypochlorite device at two amperage regimes: [a] 300 mA (130 mA·cm^{-2}, with respect to the electrode surface) and [b] 60 mA (30 mA·cm^{-2}, with respect to the electrode surface). The electrolyte was a 20% NaCl solution at 80°C. The temperature was monitored using a stainless steel thermocouple. The blue curve was computed considering Faraday's law for electrochemical reaction and represents the theoretical production at 100% current efficiency. The red curve was calculated linearly fitting the experimental point, which were retrieved using a Lovibond® CHECKIT colorimetric comparator test-kit for high range (10 – 300 mg/L of product) and collecting samples for the buffer solution. Comparing the slopes of ideal and experimental curves, we could obtain 88% and 85% $\eta_{Faraday}$ at 130 mA·cm^{-2} and 30 mA·cm^{-2}, respectively. Experiments conducted over 1 hours confirmed that those values are roughly constant over time.

The non- ideality of the measured Faradaic efficiencies is due to competitive reactions that subtract useful current, which does not generate active chlorine that can be detected. The main anodic side reactions are (a) oxygen evolution, (b) hypochlorite reaction to form chlorate, (c) hypochlorite oxidation to chlorate species and (c) hypochlorite reduction to chloride species.

a. $4OH^- \rightarrow 2H_2O + O_2 + 4e^-$

b. $2HClO + ClO^- \rightarrow ClO_3^- + 2H^+ + 2Cl^-$

c. $6ClO^- + 3H_2O \rightarrow 2ClO_3^- + 4Cl^- + 6H^+ + 1.5O_2 + 6e^-$

d. $ClO^- + H_2O + 2e^- \rightarrow Cl^- + 2OH^-$

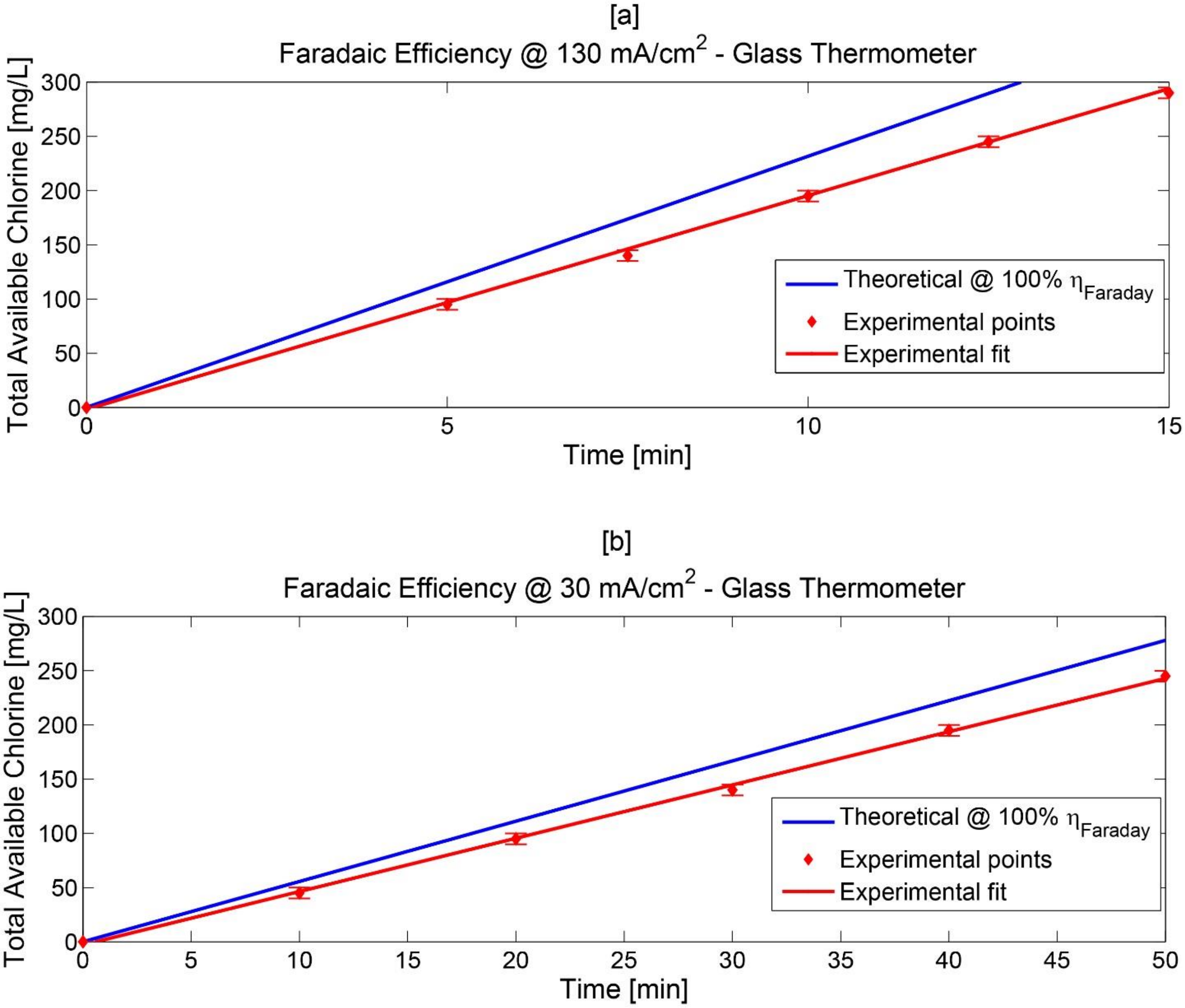

Figure S7: Faradaic efficiencies of solar-hypochlorite device at two amperage regimes: [a] 300 mA (130 mA·cm^{-2}, with respect to the electrode surface) and [b] 60 mA (30 mA·cm^{-2}, with respect to the electrode surface). The electrolyte was a 20% NaCl solution at 80°C. The temperature was monitored using a glass thermometer (purchased from VWR). The blue curve was computed considering Faraday's law for electrochemical reaction and represents the theoretical production at 100% current efficiency. The red curve was calculated linearly fitting the experimental point, which were retrieved using a Lovibond® CHECKIT colorimetric comparator test-kit for high range (10 – 300 mg/L of product) and collecting samples for the buffer solution, which were cooled before utilization. Comparing the slopes of ideal and experimental curves, we could obtain 88% and 84% $\eta_{Faraday}$ at 130 mA·cm^{-2} and 30 mA·cm^{-2}, respectively.

The comparison aimed at investigating the effect of a different temperature probe in the faradaic efficiency measurement. The stainless steel thermocouple is expected to undergo a slight corrosion when immersed in the operative electrolyte, and this could potentially affect the efficiency. Based on our experimental validation, we could conclude that the stainless steel probe has no effect of relevance in the faradaic yield determination.

Figures S6 and S7 report the total available chlorine, which measures the totality of the chlorinated species available in solution. Hypochlorite species (as well as hydrochlorous acid) will be present in liquid form; molecular chlorine species (Cl_2), if present, will tend to dissolve too, given the high solubility of chlorine in water (e.g. at 80°C, more than 2 grams of chlorine are needed to saturate 1 kg of water). Since the production is in the mg/L range, all chlorinated species will remain into the electrolyte, and thus be detected using the comparator

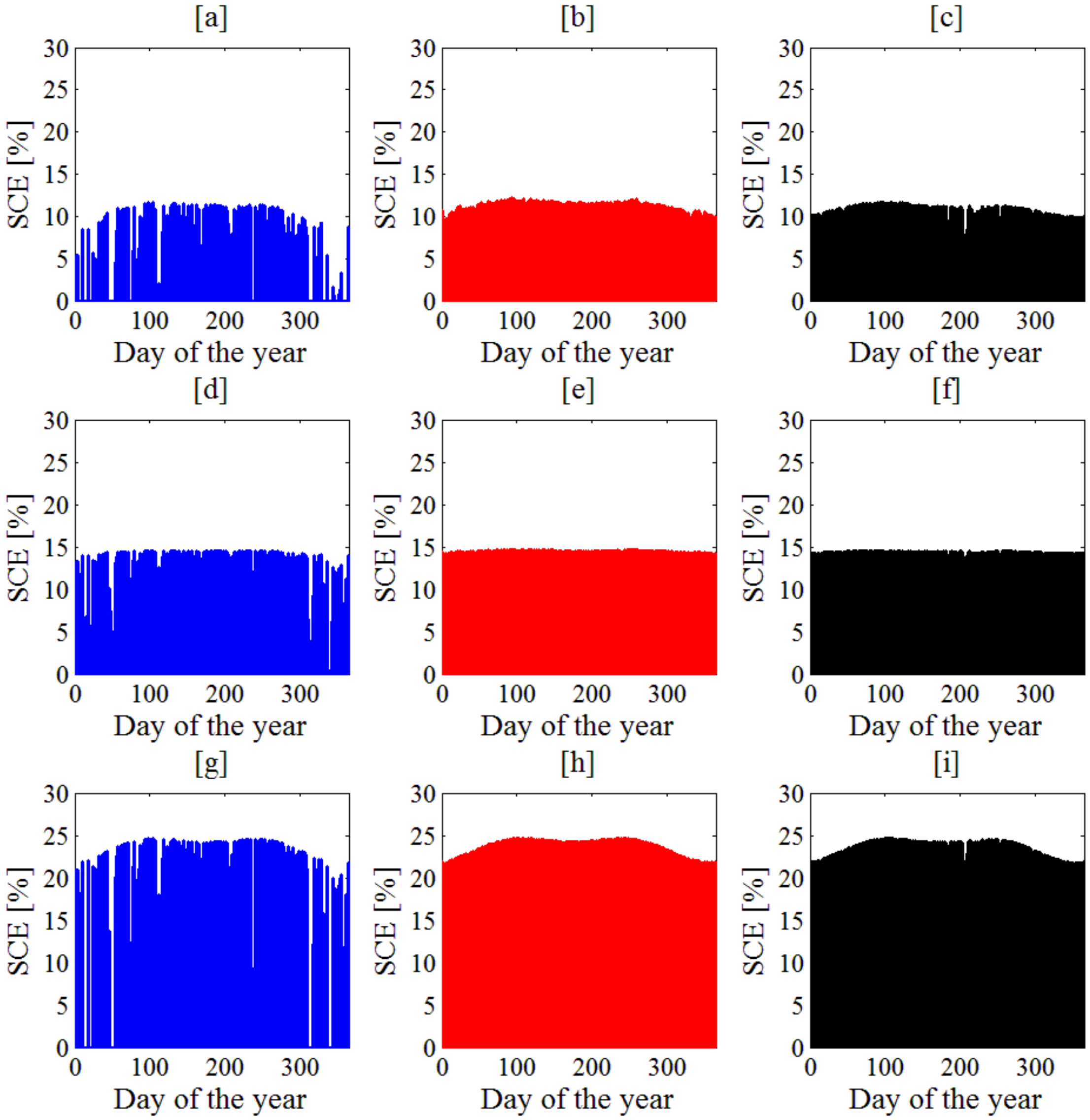

Figure S8. Solar-to-chemical conversion efficiencies (SCE) for the three PV technologies (4SHJ, 5SHJ, MJ) in three locations: Lausanne (CH), Phoenix (AZ), Delhi (IN). PV arrays are considered oriented towards the equator and having a fixed tilt: 36°, 23°, 18° for Geneva, Phoenix, Delhi, respectively. [a], [b], [c] report the SCEs for 4SHJ-powered devices throughout the year in Lausanne, Phoenix and Delhi, respectively. [d], [e], [f] report the SCEs for 5SHJ-powered devices throughout the year in Lausanne, Phoenix and Delhi, respectively. [g], [h], [i] report the SCEs for MJ-powered devices throughout the year in Lausanne, Phoenix and Delhi, respectively.

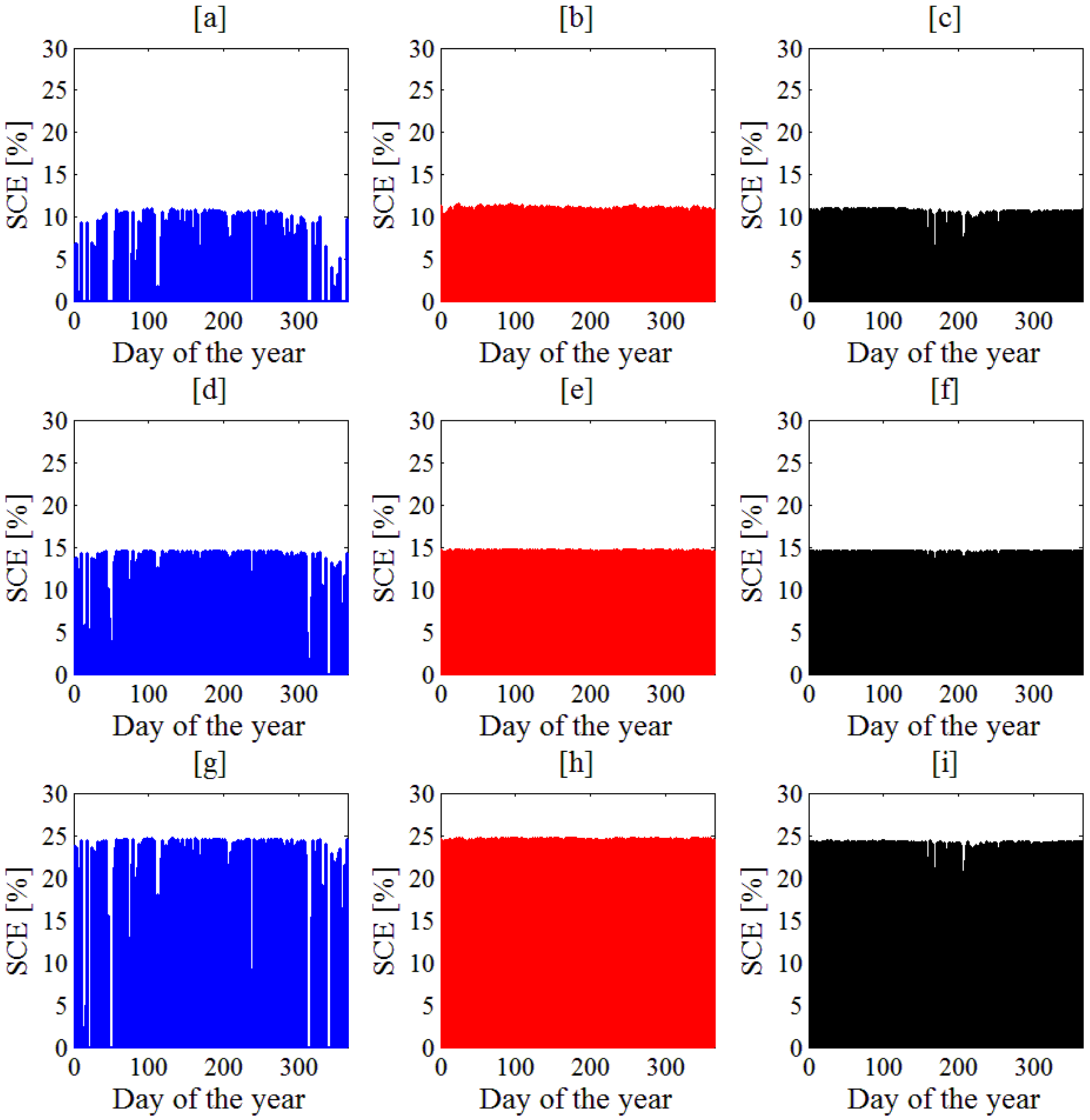

Figure S9. Solar-to-chemical conversion efficiencies (SCE) for the three PV technologies (4SHJ, 5SHJ, MJ) in three locations: Lausanne (CH), Phoenix (AZ), Delhi (IN). PV arrays are considered oriented towards the equator and having a tilt adjusted every month according to Table S2.[a], [b], [c] report the SCEs for 4SHJ-powered devices throughout the year in Lausanne, Phoenix and Delhi, respectively. [d], [e], [f] report the SCEs for 5SHJ-powered devices throughout the year in Lausanne, Phoenix and Delhi, respectively. [g], [h], [i] report the SCEs for MJ-powered devices throughout the year in Lausanne, Phoenix and Delhi, respectively.

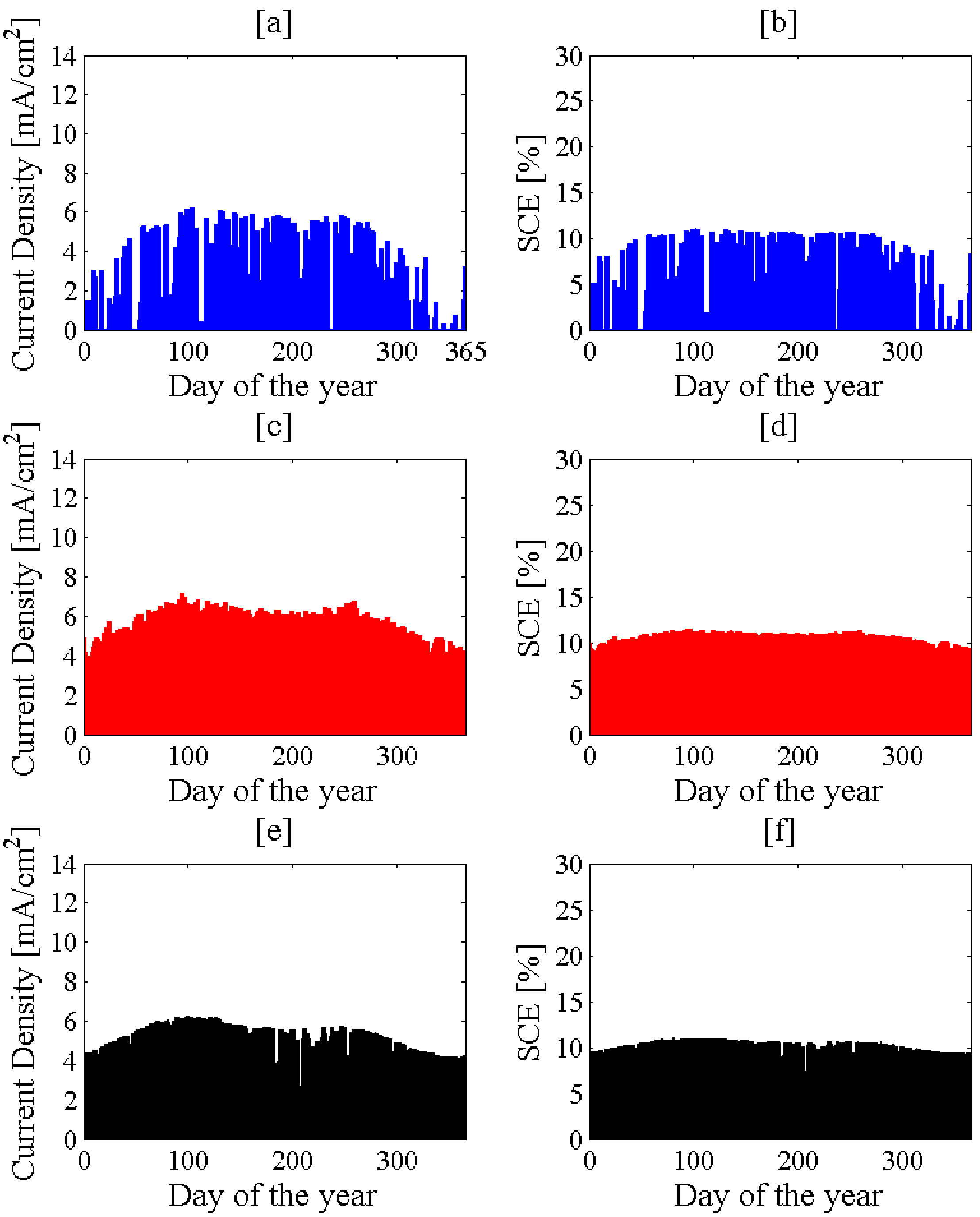

Figure S10: Working current densities [mA·cm^{-2}] and SCEs for 4SHJ-powered devices; no external tracking intervention was employed. The array tilt is fixed: 36°, 23°, 18° from the horizontal in Lausanne, Phoenix, Delhi, respectively. [a] and [b] report j and SCE for Lausanne (Switzerland); [c] and [d] report j and SCE for Phoenix (U.S.); [e] and [f] report j and SCE for Delhi (India)

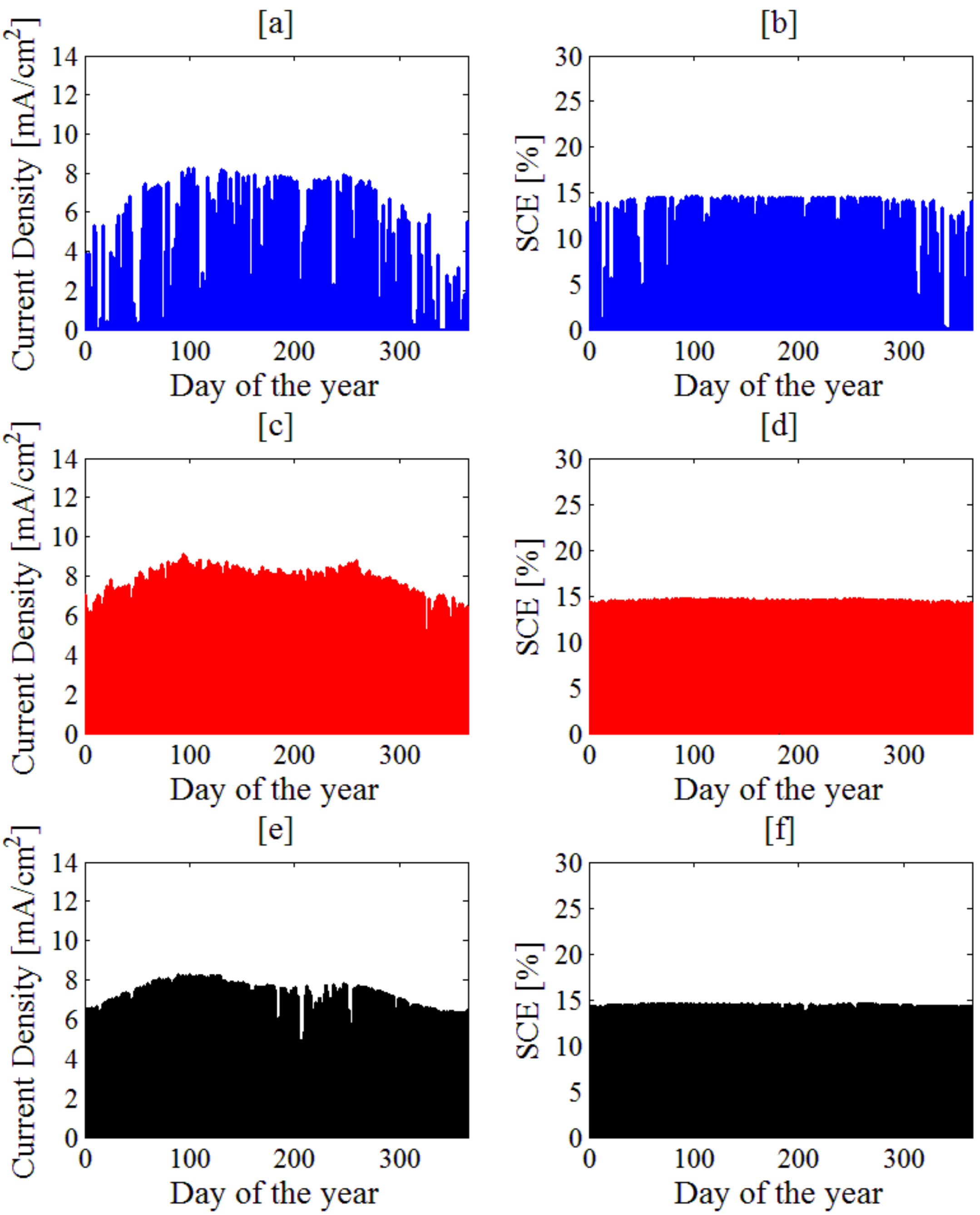

Figure S11: Working current densities [mA·cm^{-2}] and SCEs for 5SHJ-powered devices; no external tracking intervention was employed. The array tilt is fixed: 36°, 23°, 18° from the horizontal in Lausanne, Phoenix, Delhi, respectively. [a] and [b] report j and SCE for Lausanne (Switzerland); [c] and [d] report j and SCE for Phoenix (U.S.); [e] and [f] report j and SCE for Delhi (India).

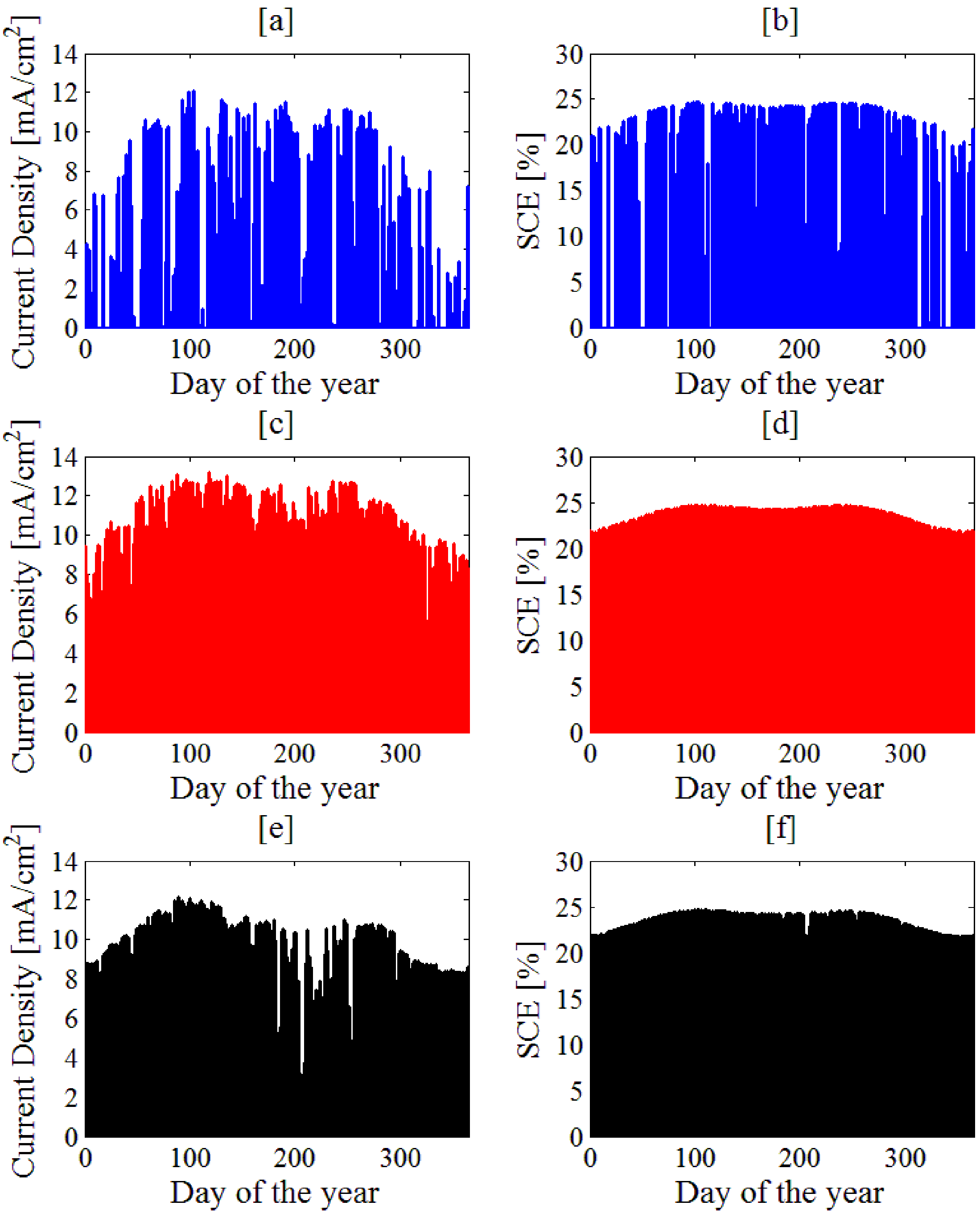

Figure S12: Working current densities [mA·cm^{-2}] and SCEs for MJ-powered devices; no external tracking intervention was employed. The array tilt is fixed: 36°, 23°, 18° from the horizontal in Lausanne, Phoenix, Delhi, respectively. [a] and [b] report j and SCE for Lausanne (Switzerland); [c] and [d] report j and SCE for Phoenix (U.S.); [e] and [f] report j and SCE for Delhi (India).

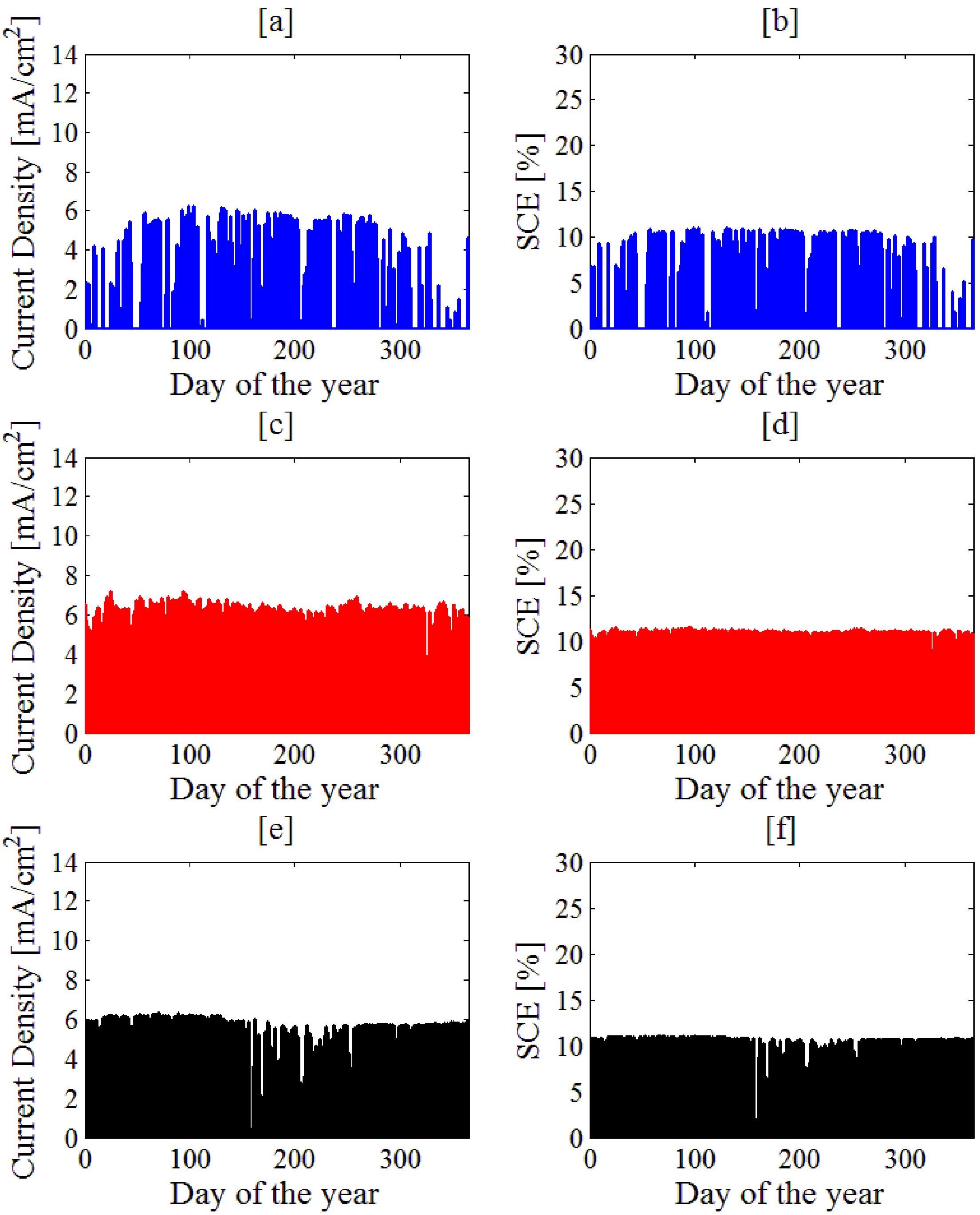

Figure S13: Working current densities [mA·cm^{-2}] and SCEs for 4SHJ-powered devices in case the array tilt is adjusted monthly according to Table S2.[a] and [b] report j and SCE for Geneva (Switzerland); [c] and [d] report j and SCE for Phoenix (U.S.); [e] and [f] report j and SCE for Delhi (India).

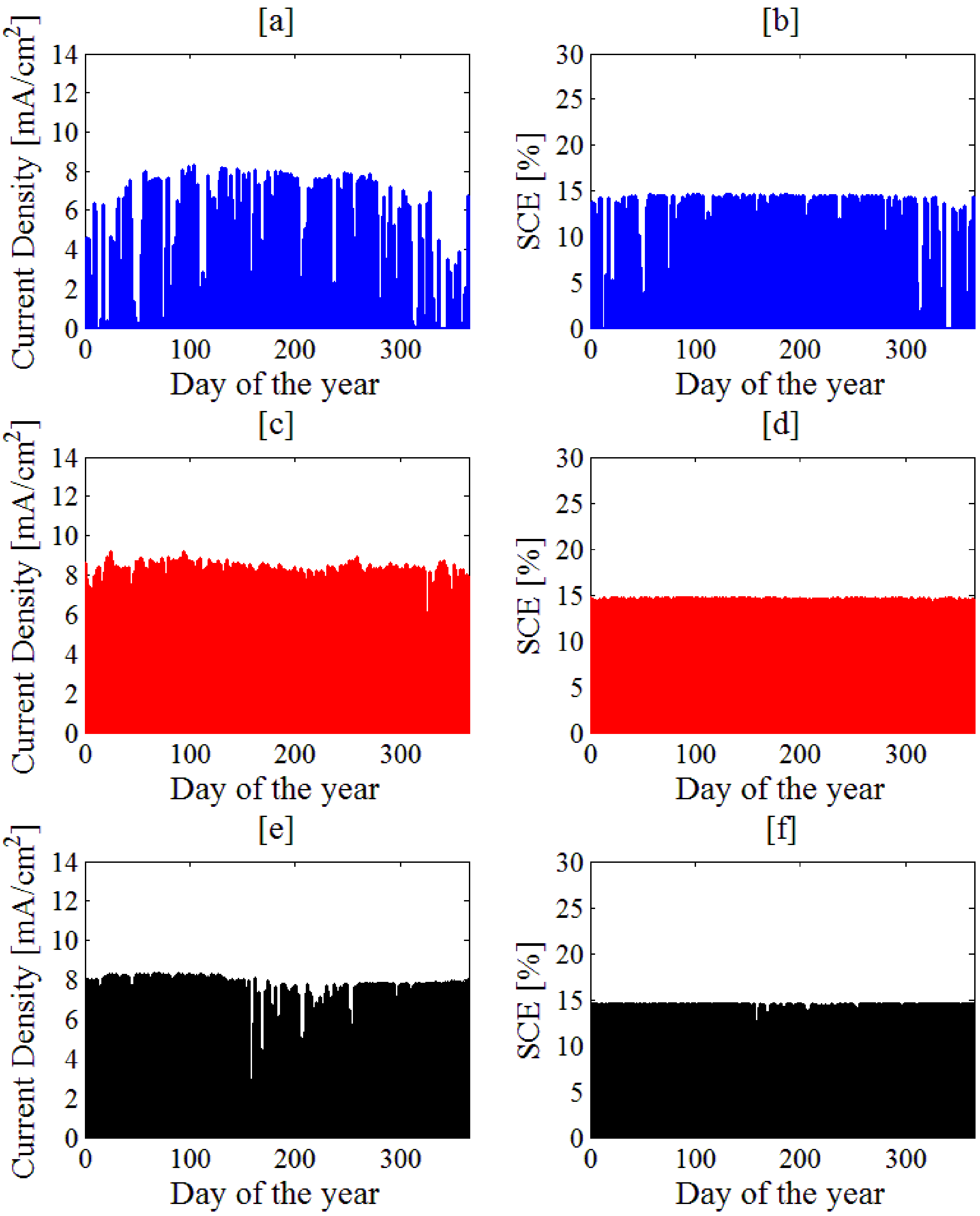

Figure S14: Working current densities [mA·cm^{-2}] and SCEs for 5SHJ-powered devices in case the array tilt is adjusted monthly according to Table S2.[a] and [b] report j and SCE for Geneva (Switzerland); [c] and [d] report j and SCE for Phoenix (U.S.); [e] and [f] report j and SCE for Delhi (India).

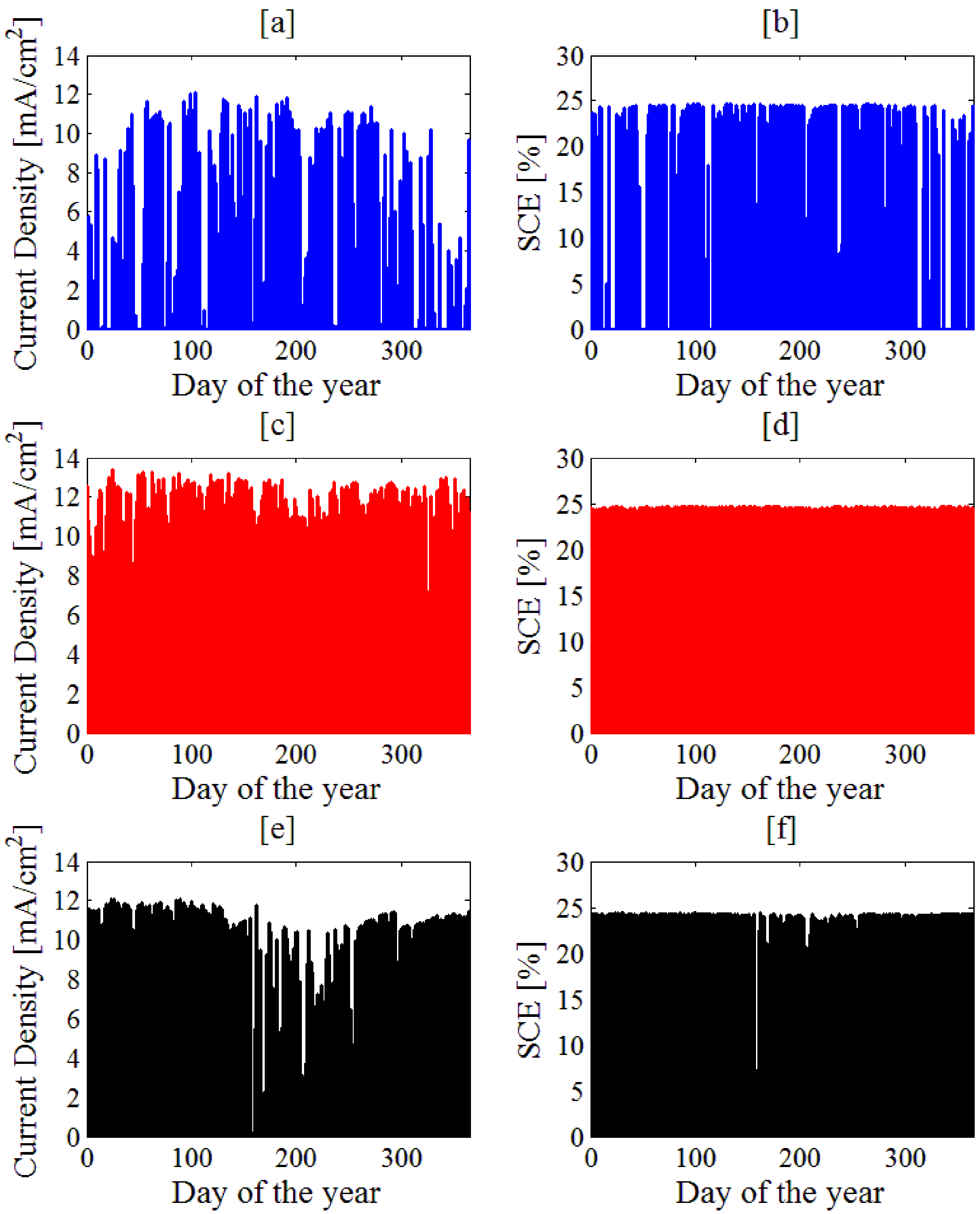

Figure S15: Working current densities [mA·cm^{-2}] and SCEs for MJ-powered devices in case the array tilt is adjusted monthly according to Table S2.[a] and [b] report j and SCE for Geneva (Switzerland); [c] and [d] report j and SCE for Phoenix (U.S.); [e] and [f] report j and SCE for Delhi (India).

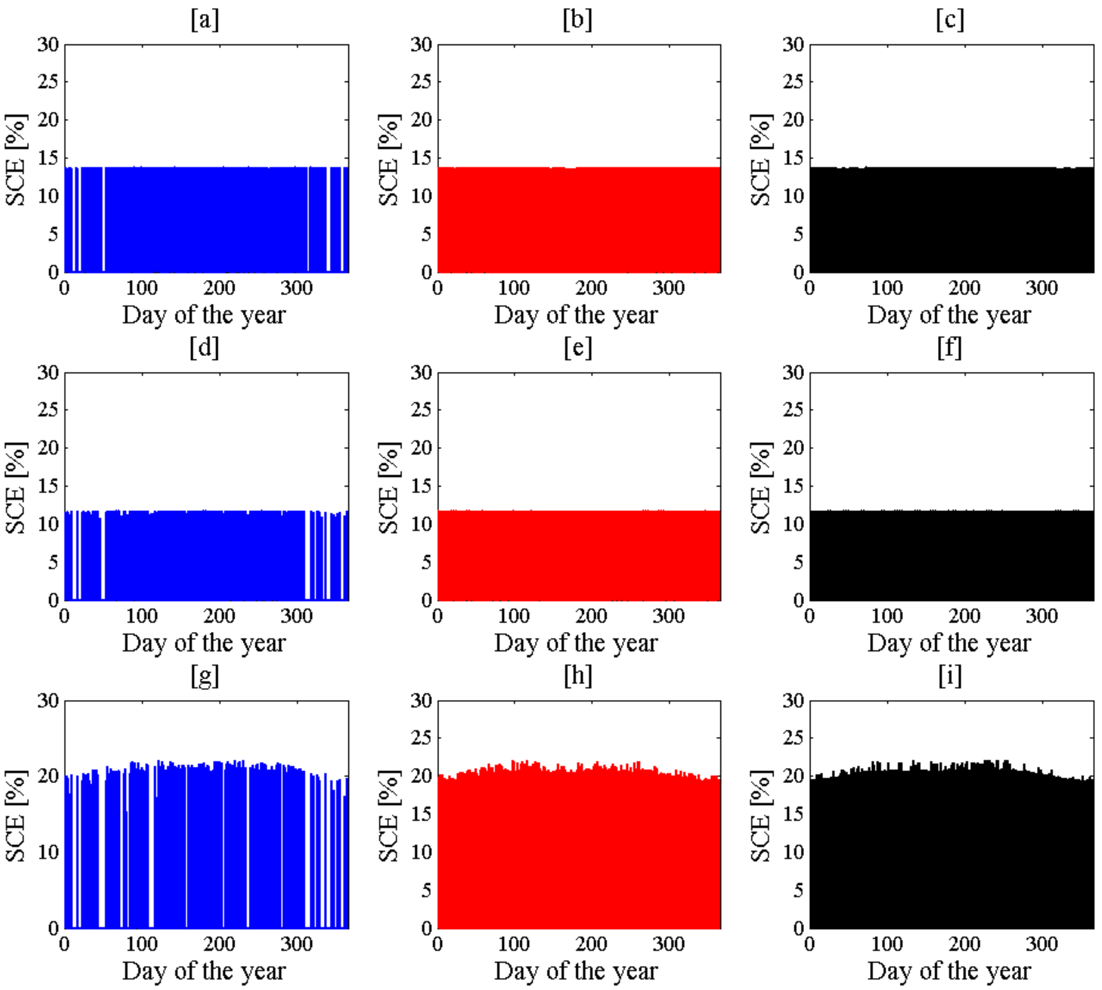

Figure S16: Solar-to-chemical conversion efficiencies (SCE) for the three PV technologies (4SHJ, 5SHJ, MJ) when a MPPT and a DC-DC converter are included in the device, in three locations: Lausanne (CH), Phoenix (AZ), Delhi (IN). PV arrays are considered oriented towards the equator and having a fixed tilt: 36°, 23°, 18° for Geneva, Phoenix, Delhi, respectively; no external tracking intervention was employed. [a], [b], [c] report the SCEs for 4SHJ-powered devices throughout the year in Lausanne, Phoenix and Delhi, respectively. [d], [e], [f] report the SCEs for 5SHJ-powered devices throughout the year in Lausanne, Phoenix and Delhi, respectively. [g], [h], [i] report the SCEs for MJ-powered devices throughout the year in Lausanne, Phoenix and Delhi, respectively.

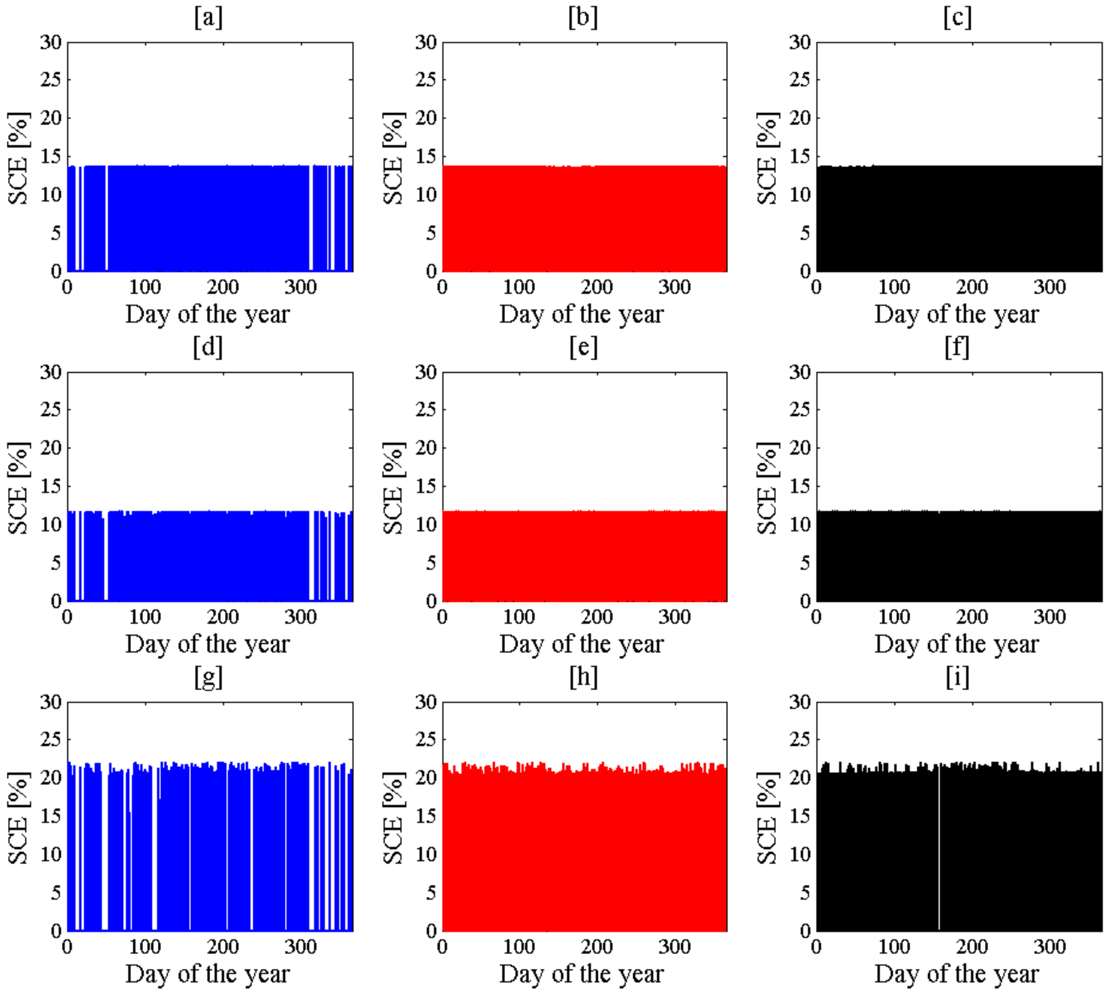

Figure S17: Solar-to-chemical conversion efficiencies (SCE) for the three PV technologies (4SHJ, 5SHJ, MJ) when a MPPT and a DC-DC converter are included in the device, in three locations: Lausanne (CH), Phoenix (AZ), Delhi (IN). PV arrays are considered oriented towards the equator and the tilt is externally adjusted each month according to Table S2.[a], [b], [c] report the SCEs for 4SHJ-powered devices throughout the year in Lausanne, Phoenix and Delhi, respectively. [d], [e], [f] report the SCEs for 5SHJ-powered devices throughout the year in Lausanne, Phoenix and Delhi, respectively. [g], [h], [i] report the SCEs for MJ-powered devices throughout the year in Lausanne, Phoenix and Delhi, respectively.

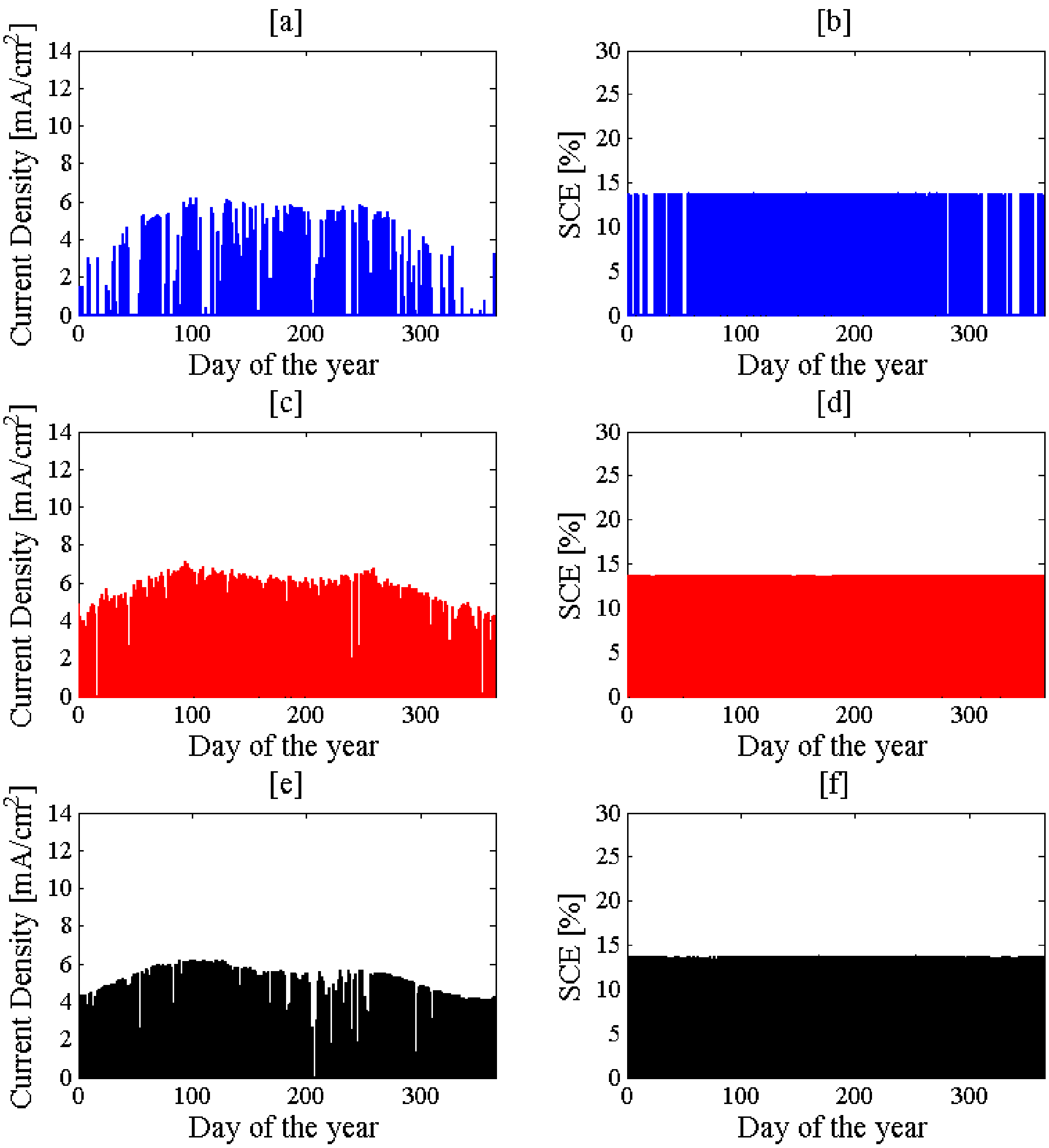

Figure S18: Working current densities [mA·cm^{-2}] and SCEs for 4SHJ-powered devices in case a MPPT coupled with a DC-DC converter is introduced in the system; no external tracking intervention was implemented. PV arrays are considered oriented towards the equator and tilted of 36°, 23°, 18° with respect to the horizontal in Lausanne, Phoenix and Delhi, respectively. [a] and [b] report j and SCE for Lausanne (Switzerland); [c] and [d] report j and SCE for Phoenix (U.S.); [e] and [f] report j and SCE for Delhi (India).

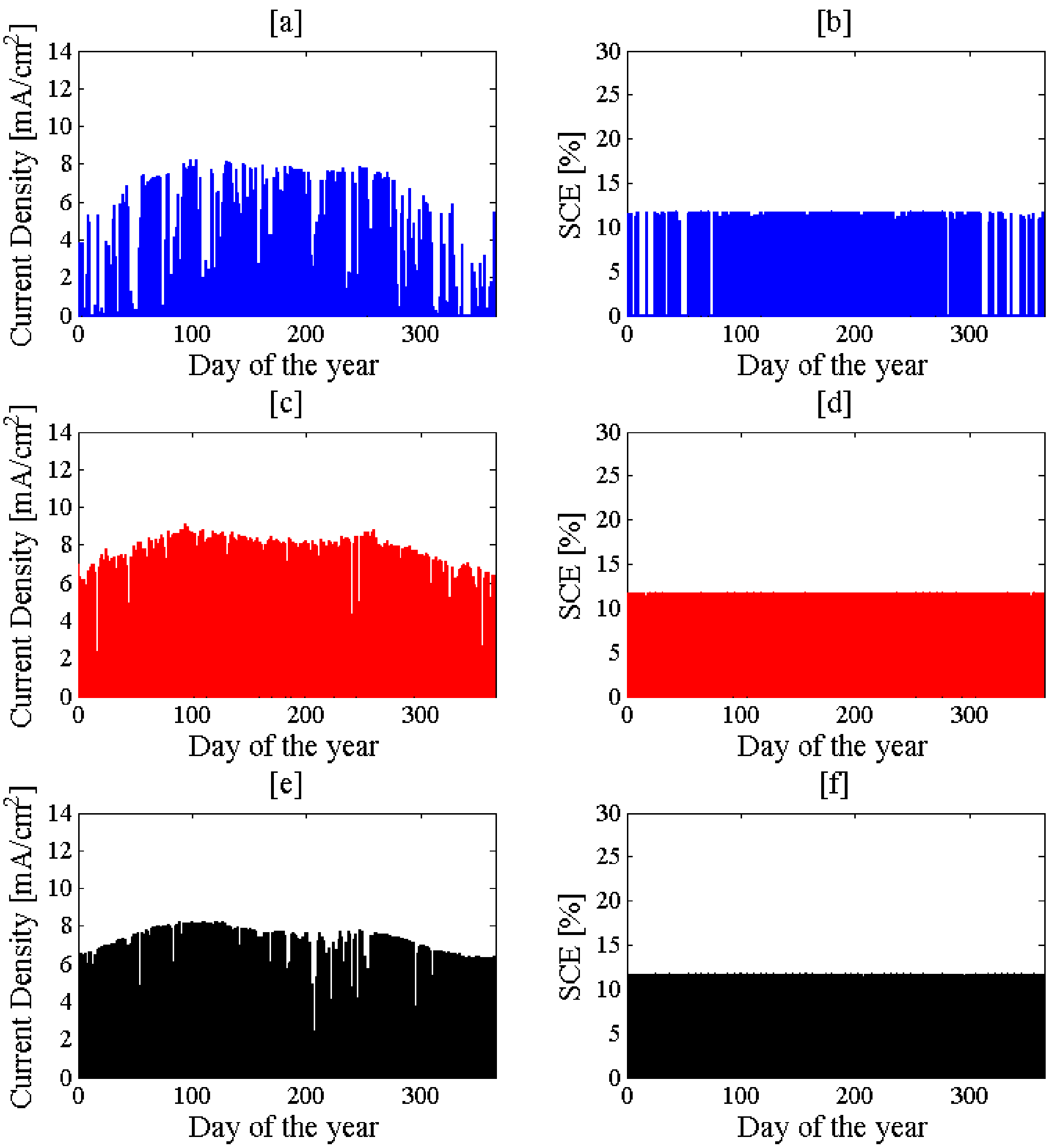

Figure S19: Working current densities [mA·cm^{-2}] and SCEs for 5SHJ-powered devices in case a MPPT coupled with a DC-DC converter is introduced in the system; no external tracking intervention was implemented. PV arrays are considered oriented towards the equator and tilted of 36°, 23°, 18° with respect to the horizontal in Lausanne, Phoenix and Delhi, respectively. [a] and [b] report j and SCE for Lausanne (Switzerland); [c] and [d] report j and SCE for Phoenix (U.S.); [e] and [f] report j and SCE for Delhi (India).

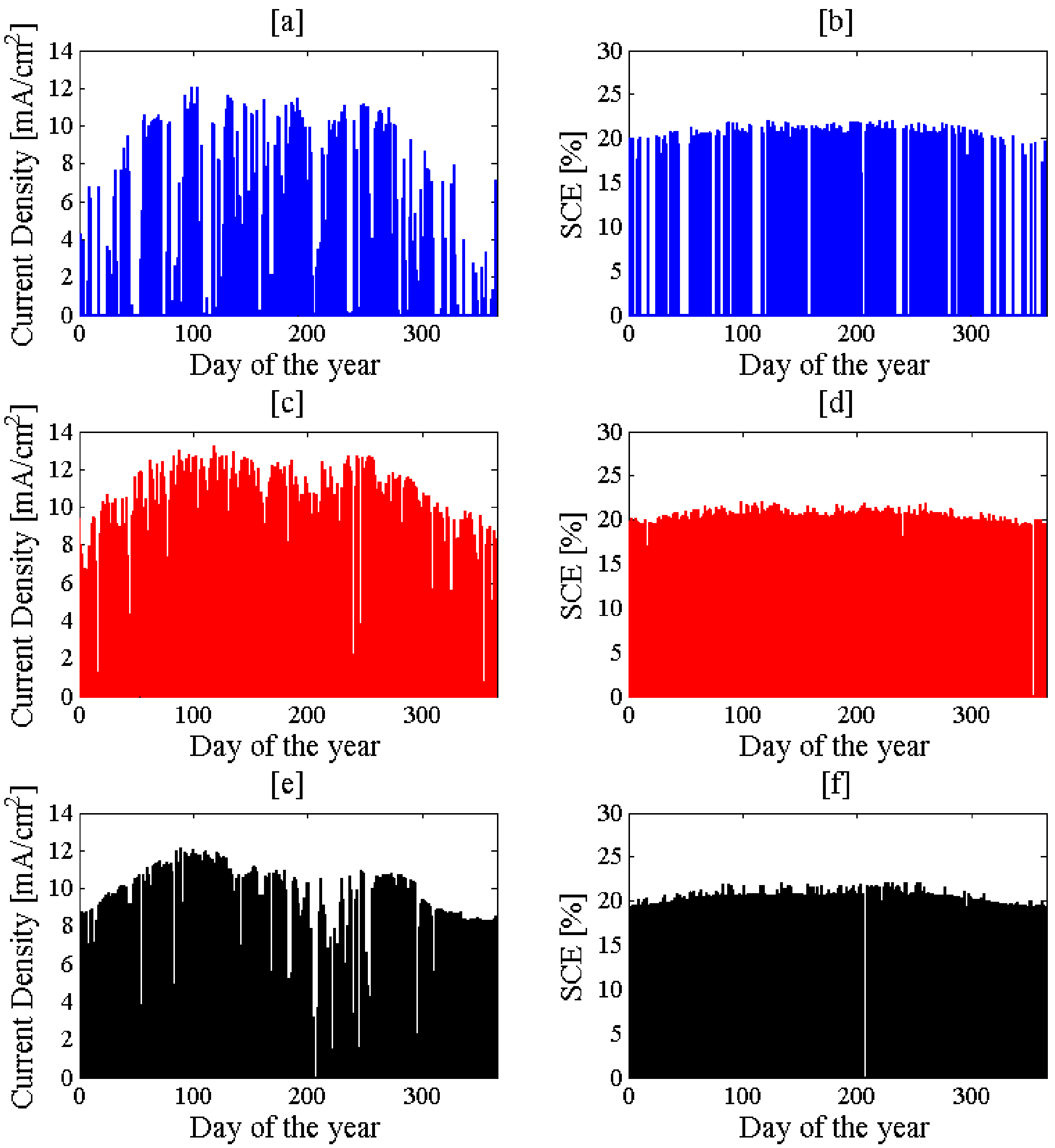

Figure S20: Working current densities [mA·cm^{-2}] and SCEs for MJ-powered devices in case a MPPT coupled with a DC-DC converter is introduced in the system; no external tracking intervention was implemented. PV arrays are considered oriented towards the equator and tilted of 36°, 23°, 18° with respect to the horizontal in Lausanne, Phoenix and Delhi, respectively. [a] and [b] report j and SCE for Lausanne (Switzerland); [c] and [d] report j and SCE for Phoenix (U.S.); [e] and [f] report j and SCE for Delhi (India).

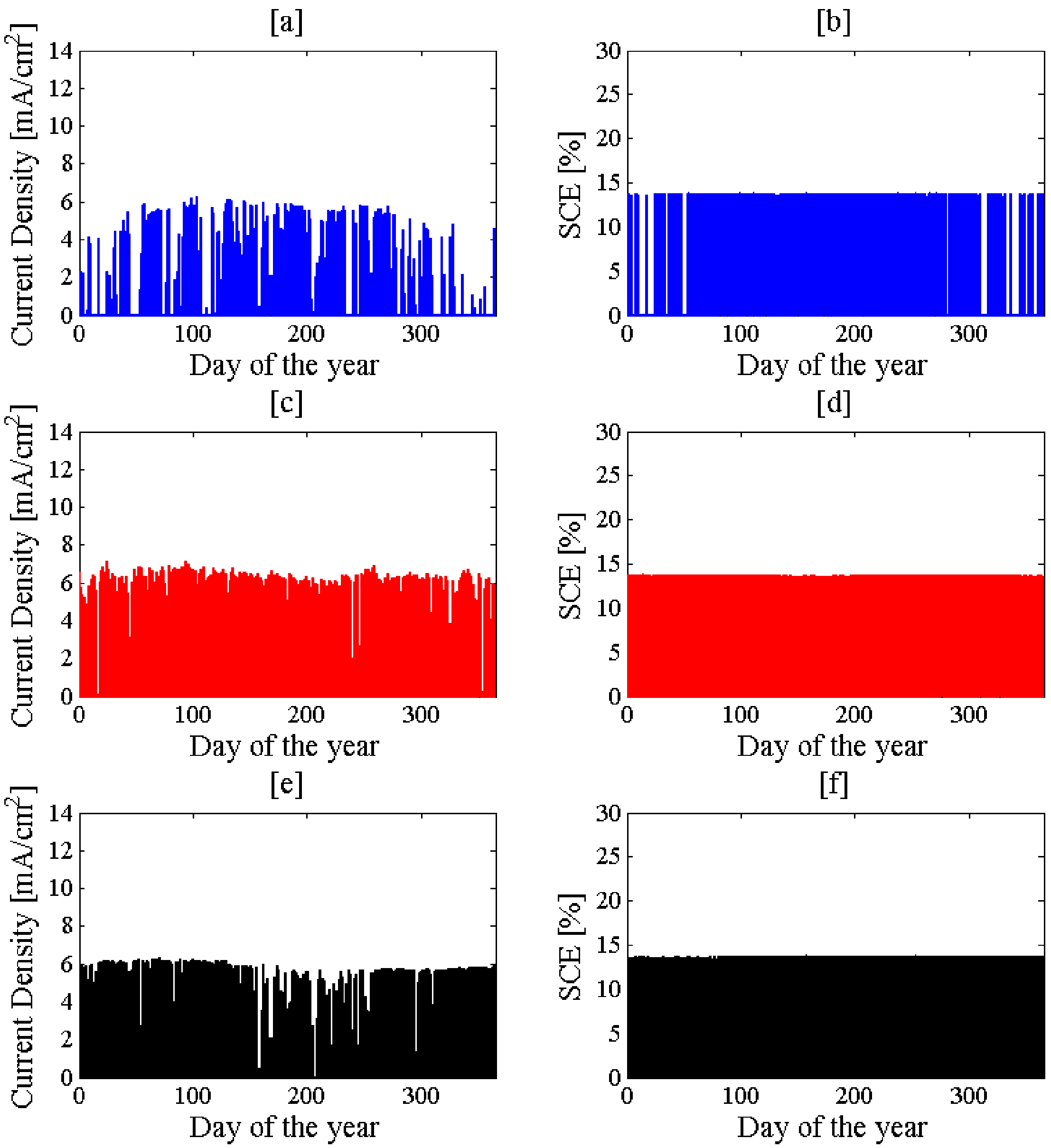

Figure S21: Working current densities [mA·cm^{-2}] and SCEs for 4SHJ-powered devices in case a MPPT coupled with a DC-DC converter is introduced in the system. PV arrays are considered oriented towards the equator; the tilt is adjusted monthly according to Table S2.[a] and [b] report j and SCE for Lausanne (Switzerland); [c] and [d] report j and SCE for Phoenix (U.S.); [e] and [f] report j and SCE for Delhi (India).

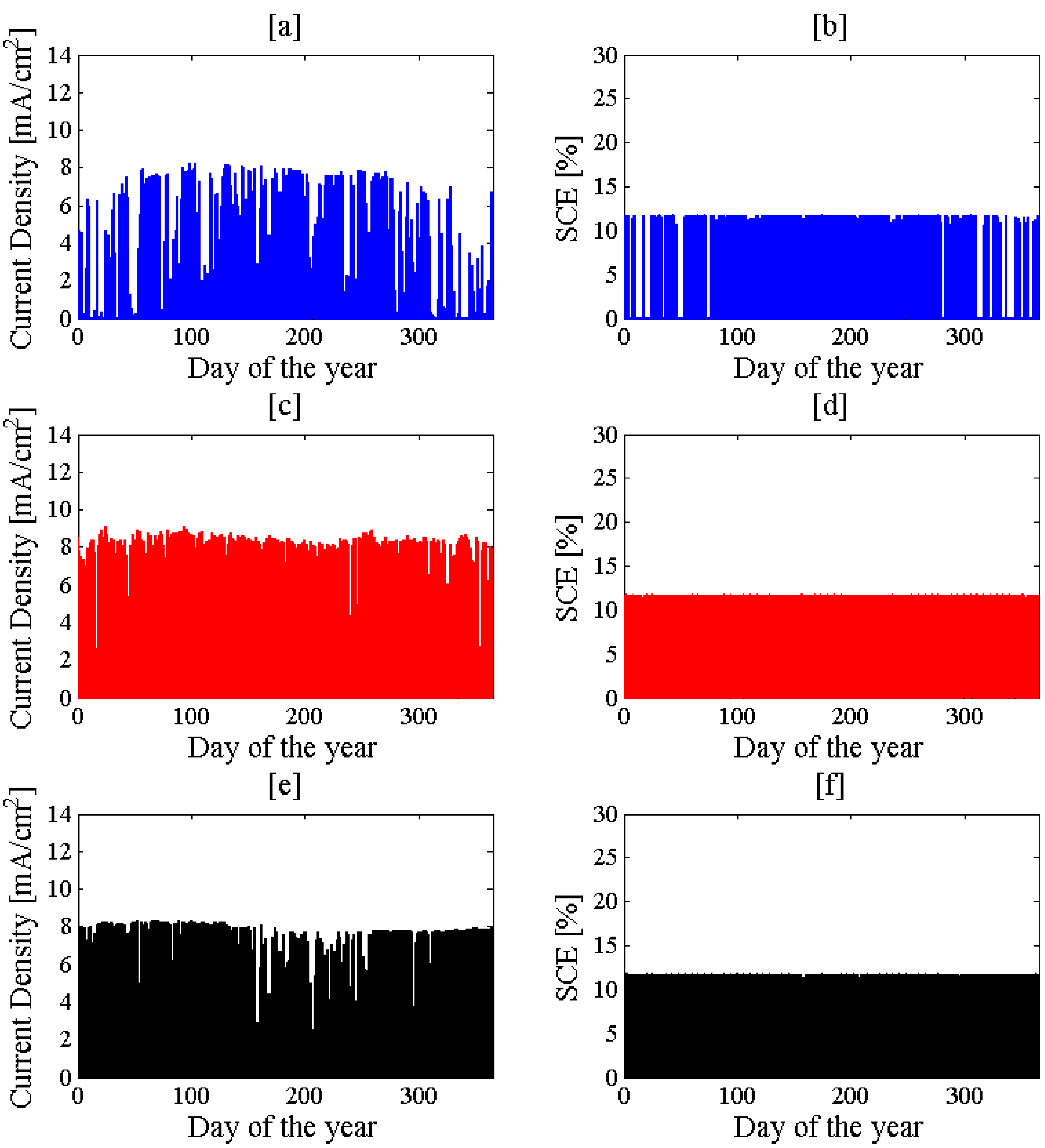

Figure S22: Working current densities [mA·cm^{-2}] and SCEs for 5SHJ-powered devices in case a MPPT coupled with a DC-DC converter is introduced in the system. PV arrays are considered oriented towards the equator; the tilt is adjusted monthly according to Table S2.[a] and [b] report j and SCE for Lausanne (Switzerland); [c] and [d] report j and SCE for Phoenix (U.S.); [e] and [f] report j and SCE for Delhi (India).

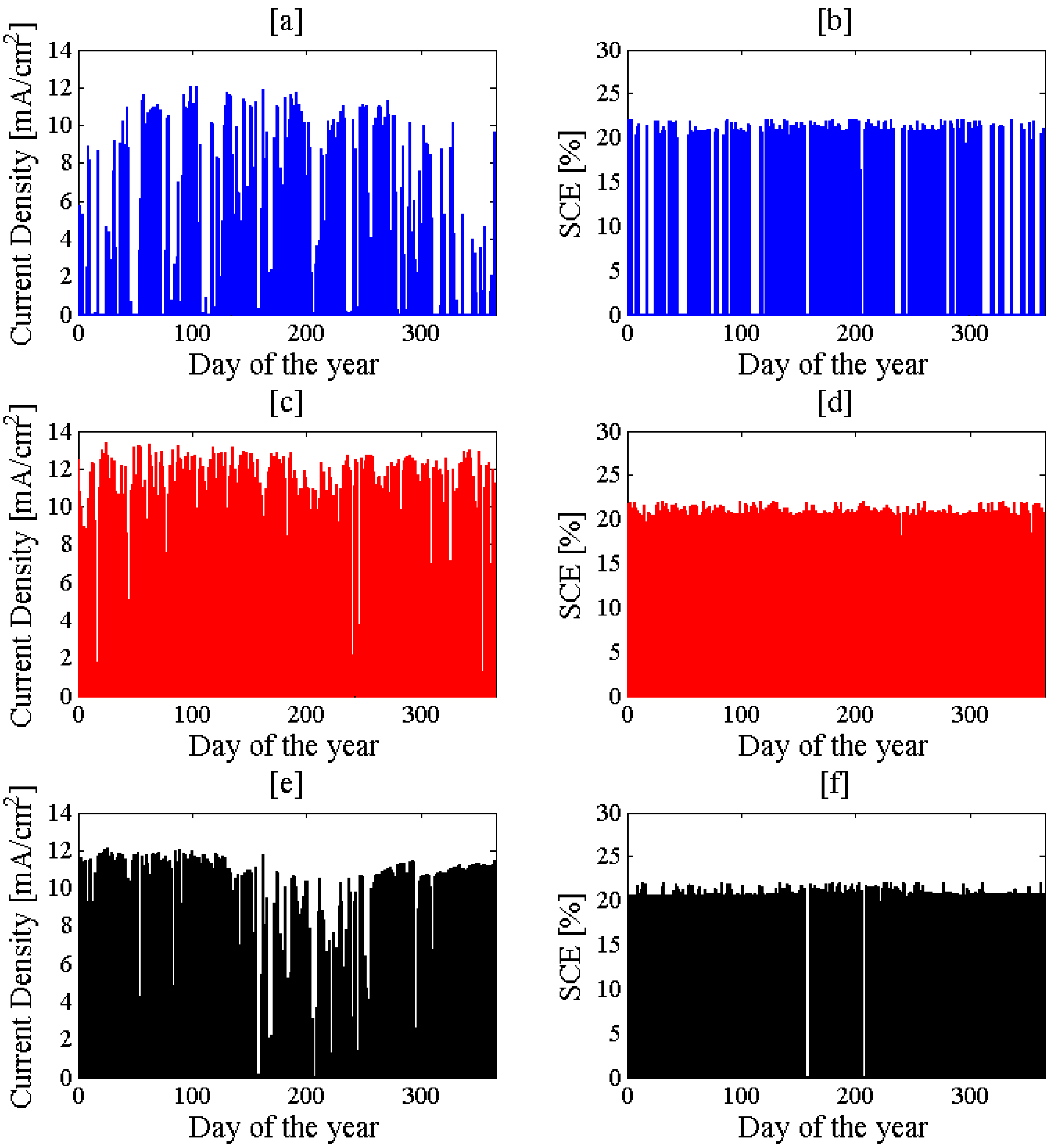

Figure S23: Working current densities [mA·cm^{-2}] and SCEs for MJ-powered devices in case a MPPT coupled with a DC-DC converter is introduced in the system. PV arrays are considered oriented towards the equator; the tilt is adjusted monthly according to Table S2.. [a] and [b] report j and SCE for Lausanne (Switzerland); [c] and [d] report j and SCE for Phoenix (U.S.); [e] and [f] report j and SCE for Delhi (India).

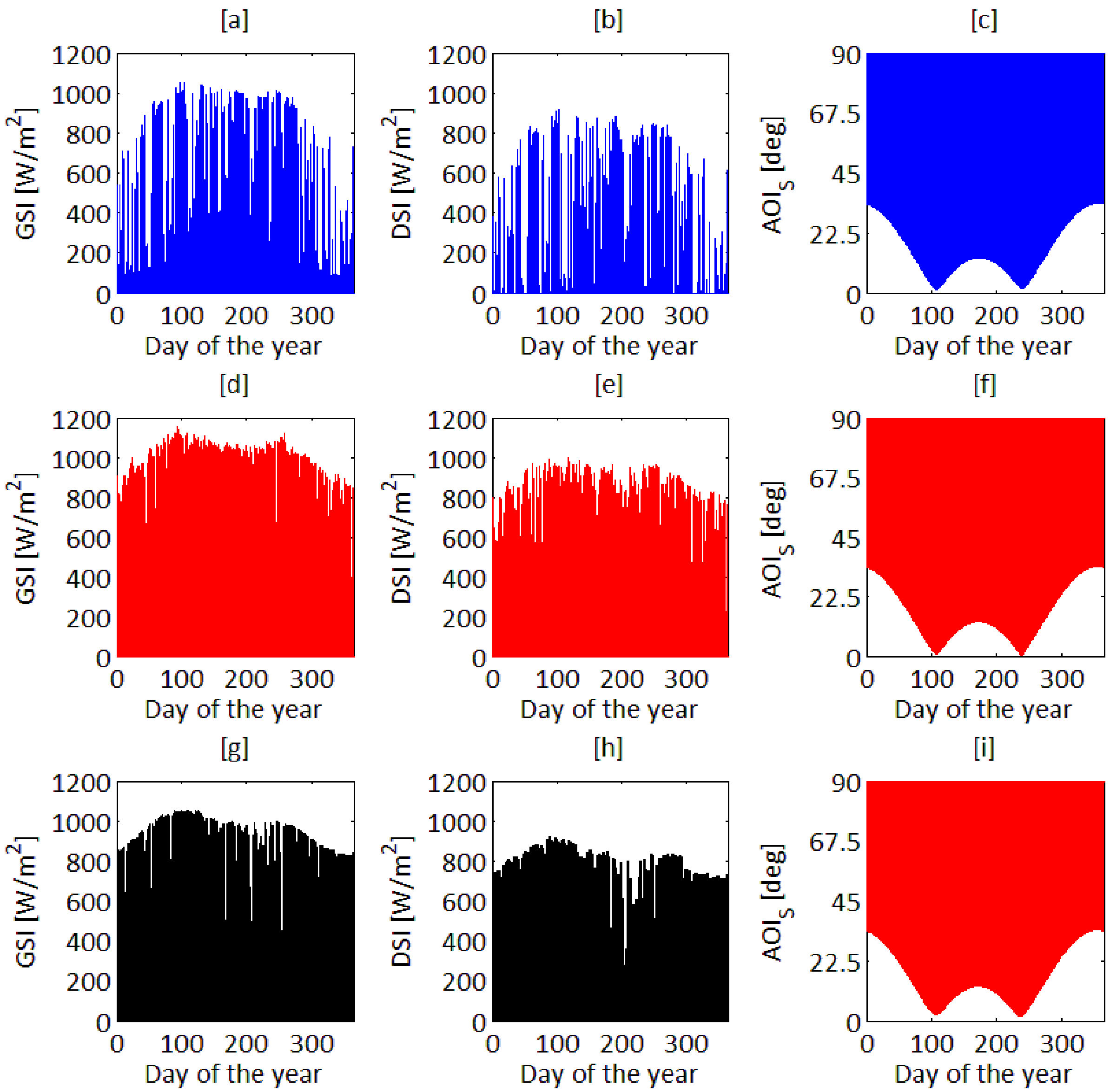

Figure S24: Solar irradiance and angle of incidence for PV surface, fixed tilt. [a], [b], [c] report the global irradiance (GSI), direct irradiance (DSI) and angle of incidence (AOI_S) for the surface exposed in Lausanne (CH), oriented south and 36° tilted with respect to the horizontal. [d], [e], [f] report the global irradiance (GSI), direct irradiance (DSI) and angle of incidence (AOI_S) for the surface exposed in Phoenix (AZ), oriented south and 23° tilted with respect to the horizontal. [g], [h], [i] report the global irradiance (GSI), direct irradiance (DSI) and angle of incidence (AOI_S) for the surface exposed in Delhi (IN), oriented south and 18° tilted with respect to the horizontal.

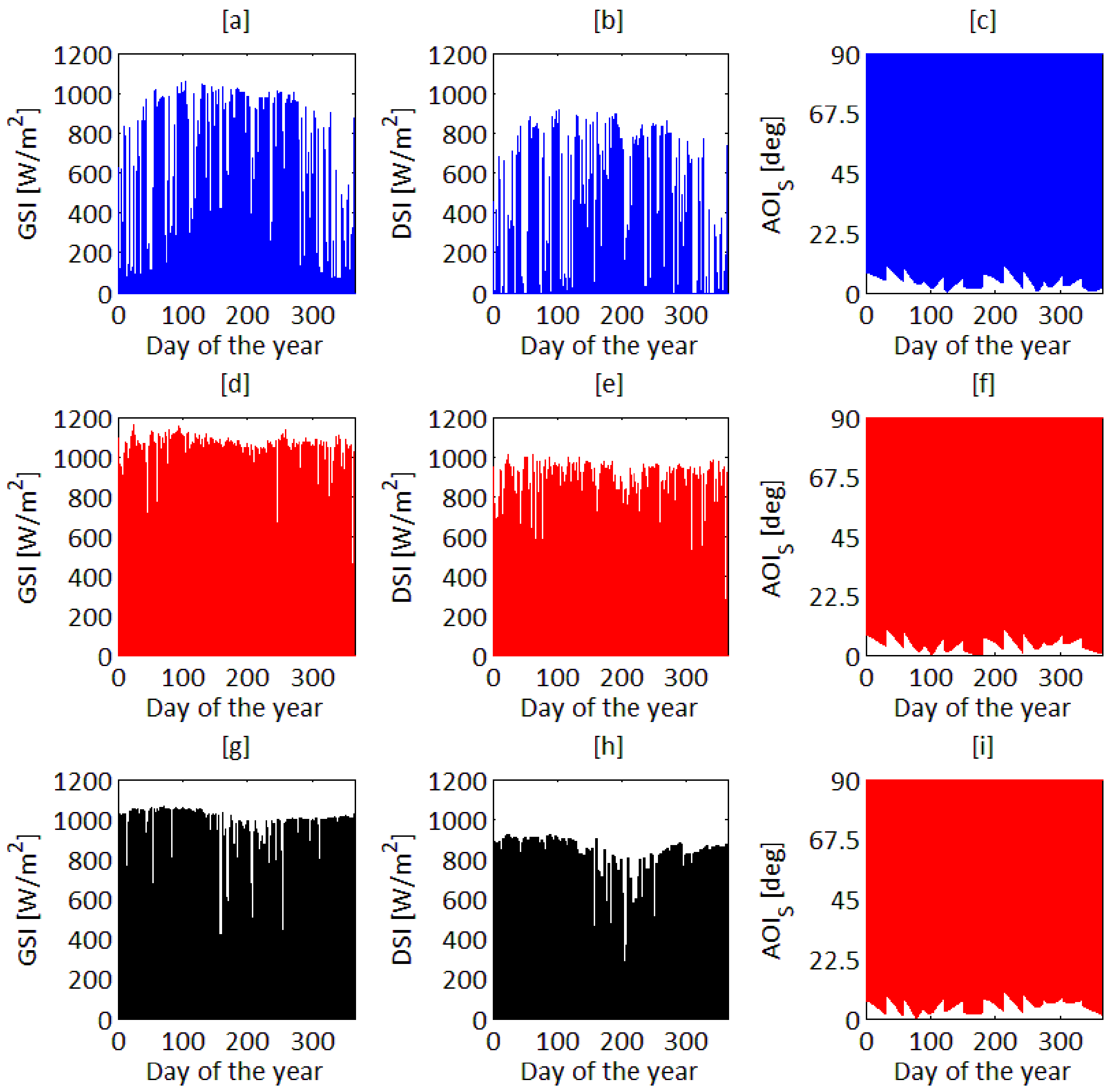

Figure S25: Solar irradiance and angle of incidence for PV surface, tilt adjusted monthly according to Table S2.[a], [b], [c] report the global irradiance (GSI), direct irradiance (DSI) and angle of incidence (AOI_S) for the surface exposed in Lausanne (CH), oriented south. [d], [e], [f] report the global irradiance (GSI), direct irradiance (DSI) and angle of incidence (AOI_S) for the surface exposed in Phoenix (AZ), oriented south. [g], [h], [i] report the global irradiance (GSI), direct irradiance (DSI) and angle of incidence (AOI_S) for the surface exposed in Delhi (IN), oriented south.

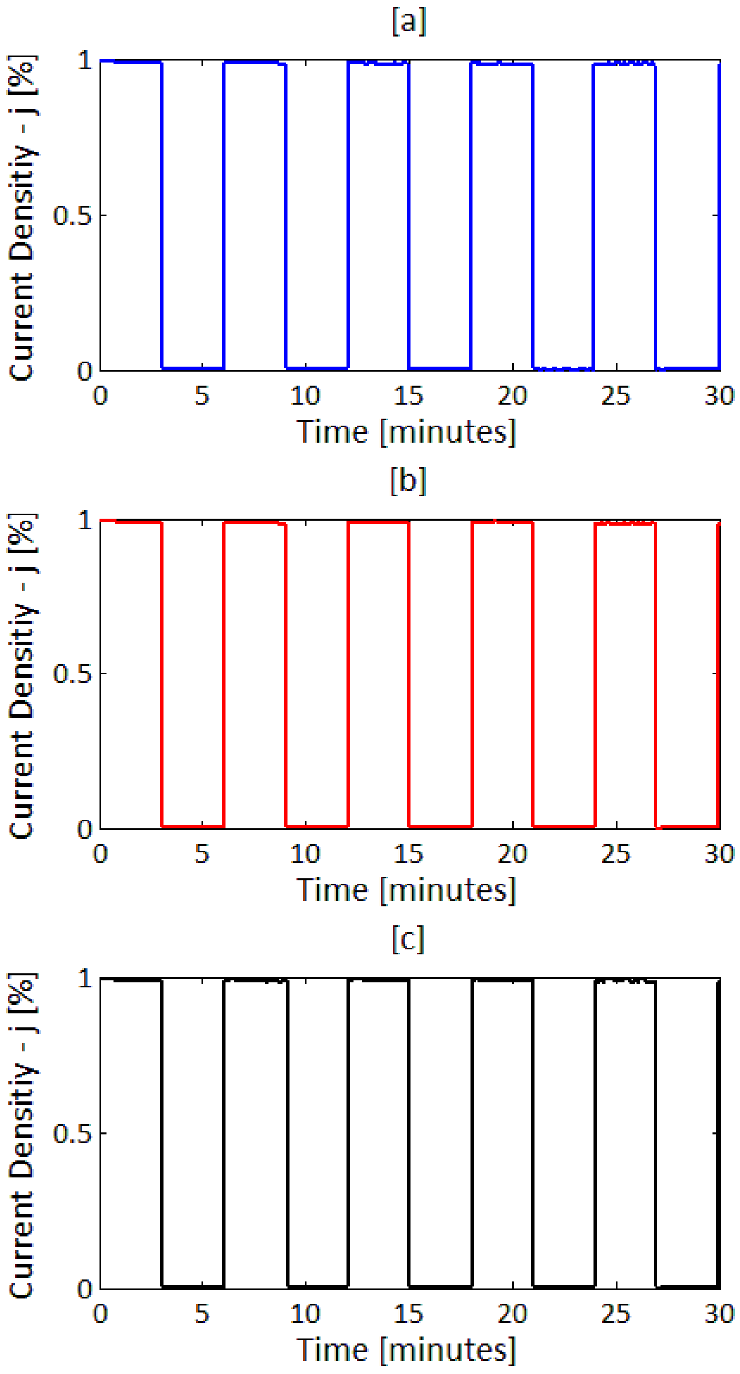

Figure S26: Relative working current densities [%] for ON-OFF input illumination. Values were normalized with respect to the maximum for each measurement. A ScienceTech SF-300 AAA class solar simulator was employed as illumination source; the beam was alternatively switched ON and OFF every 180 seconds. [a] reports the results for the 4SHJ-powered device. [b] reports the results for the 5SHJ-powered device. [c] reports the results for the MJ-powered device. No significant performance degradation effect was observed in any of the experiments.

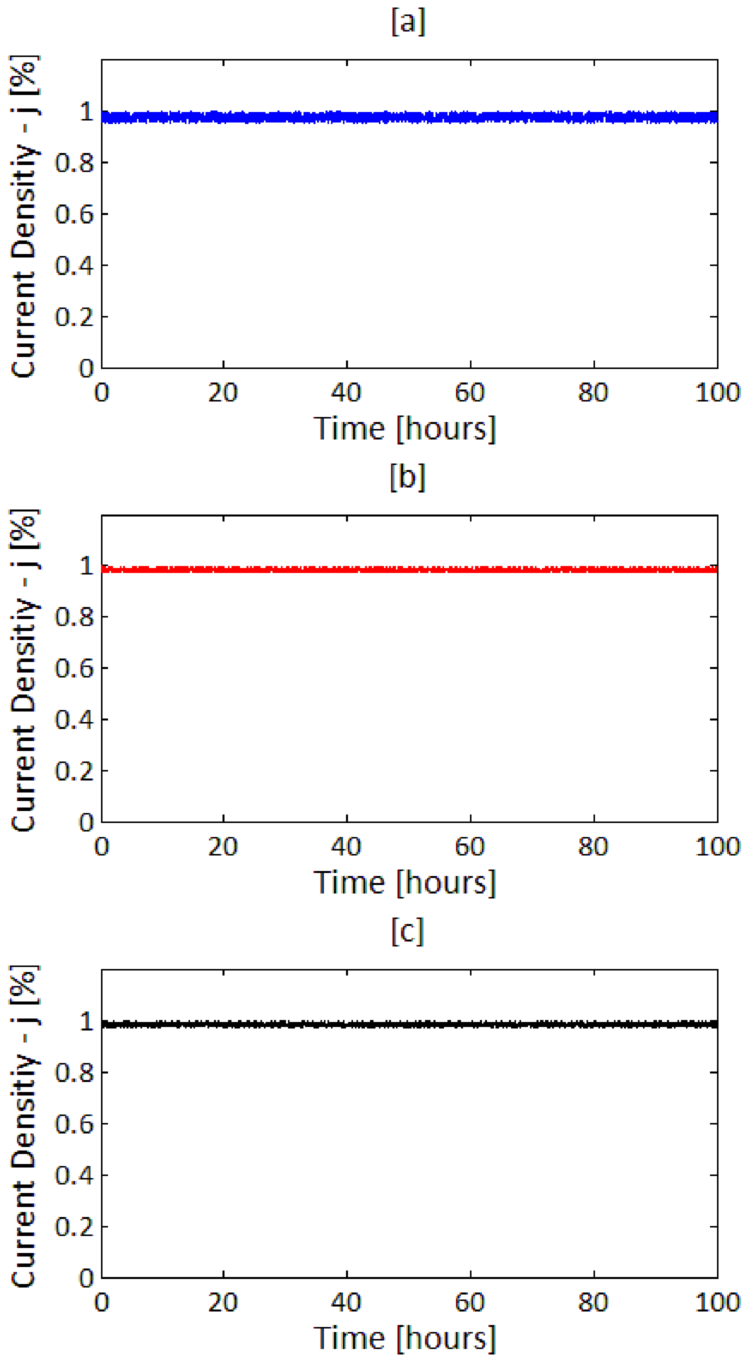

Figure S27: Relative working current densities [%] for a continuous 100 hours illumination experiment. Values were normalized with respect to the maximum for each measurement. A ScienceTech SF-300 AAA class solar simulator was employed as illumination source. The electrolyte was a 20%wt NaCl solution (80°C, temperature monitored using a steel thermocouple). [a] reports the results for the 4SHJ-powered device (Normalized mean value: 0.973; normalized standard deviation: 0.012). [b] reports the results for the 5SHJ-powered device. (Normalized mean value: 0.981; normalized standard deviation: 0.006). [c] reports the results for the MJ-powered device (Normalized mean value: 0.983; normalized standard deviation: 0.006). No significant performance degradation effect was observed in any of the experiments.

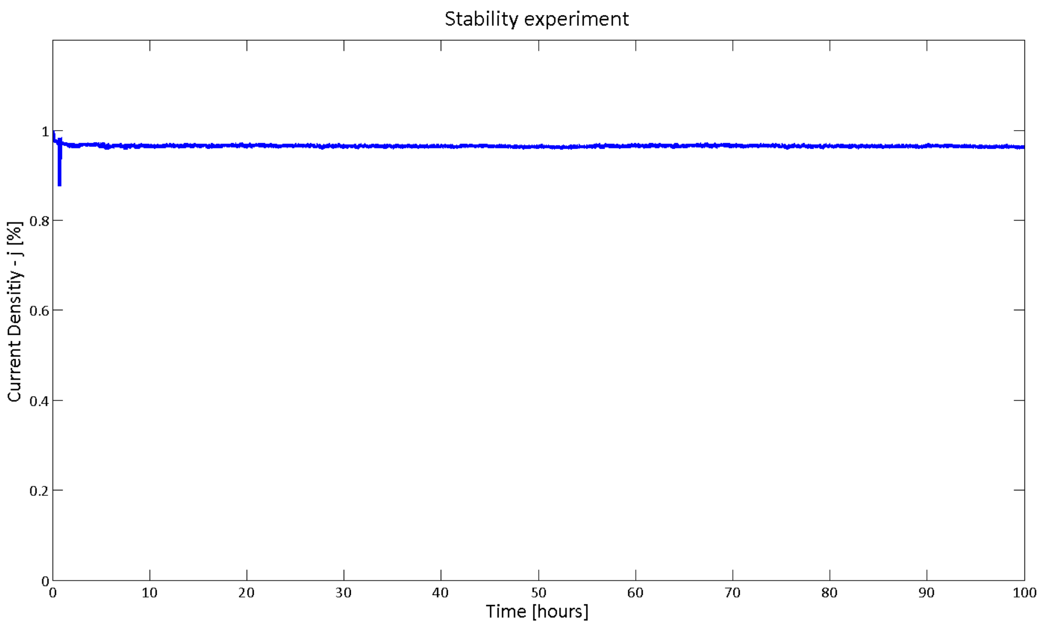

Figure S28: Relative working current densities [%] for a continuous 100 hours illumination experiment. The 5SHJ minimodule was employed in this experiment. Values were normalized with respect to the maximum. A ScienceTech SF-300 AAA class solar simulator was employed as illumination source. The electrolyte was a 20%wt NaCl solution (80°C, temperature monitored using a glass thermometer rather than a steel thermocouple).

Since the stainless steel thermocouple is likely to undergo a slight corrosion process when immersed in the electrolyte, the experiment aimed at evaluating the differences in the long term stability when replacing the mean to monitor the temperature. The outcomes showed no significant difference.

Normalized mean value: 0.975; normalized standard deviation: 0.003.

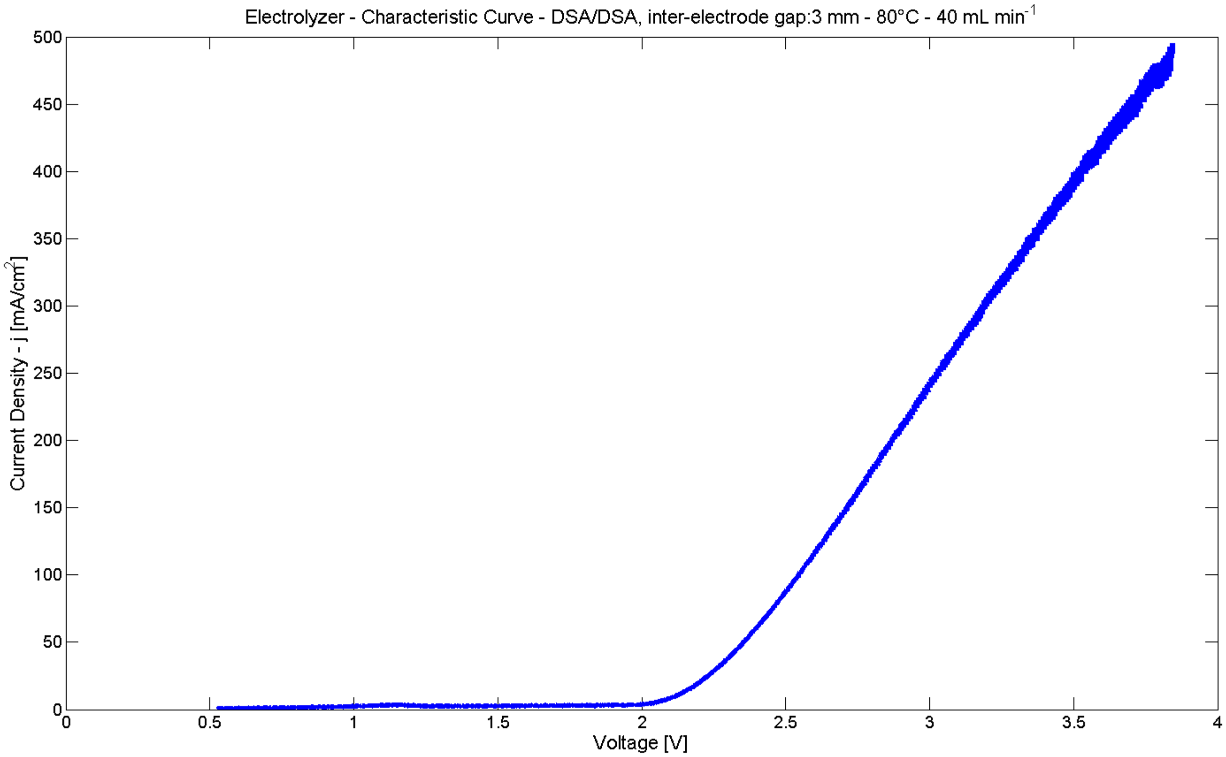

Figure S29: Characteristic curve (Current Density vs Potential) of the electrolyzer employed. The current density is here referred to the electrode area; in the main manuscript it was referred to the photovoltaic area. The assembly consisted of two DSA electrodes (Ø = 1.7 cm; inter-electrode gap 3 mm). The electrolyte was 20%wt NaCl solution, heated at 80°C and pumped inside the reactor at a flow rate of 40 mL/min. The curve highlights that the working current density is 250 mA/cm^2 at 3V. This is consistent with values in literature.

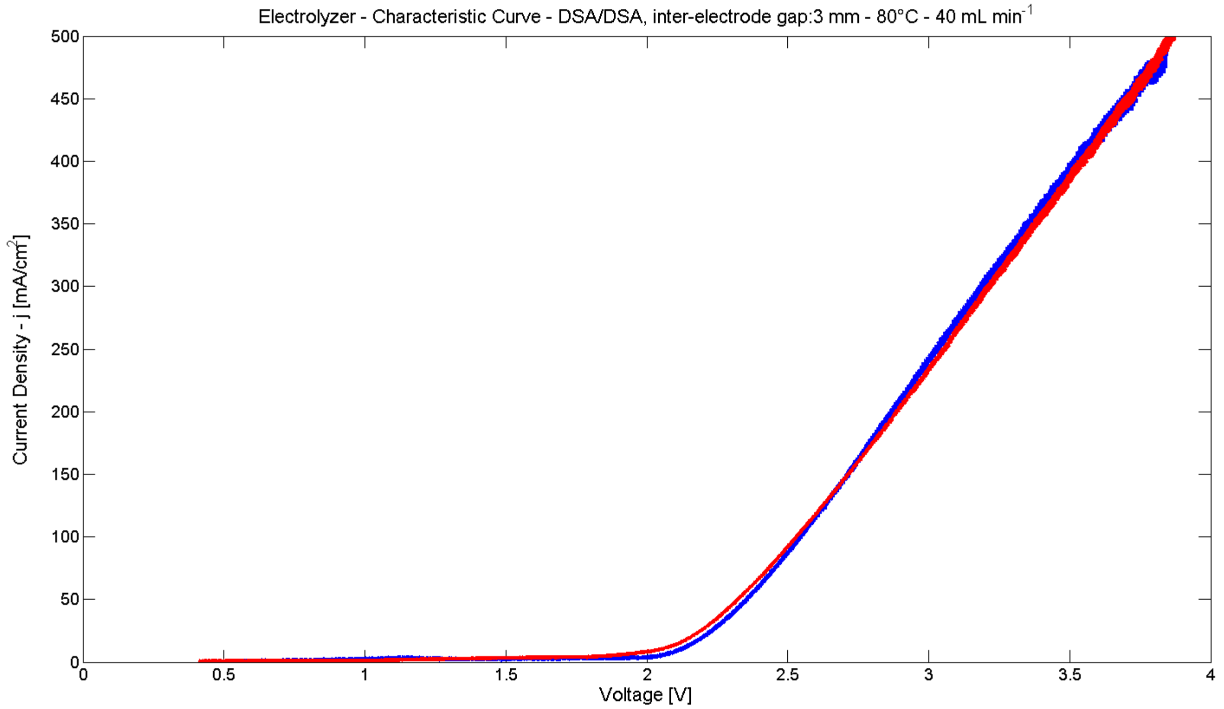

Figure S30: Characteristic curve (Current Density vs Potential) of the electrolyzer employed with two different temperature probes. The blue curve refers to the case in which the electrolyte batch temperature was monitored with a stainless steel thermocouple; the red curve refers to the case in which the electrolyte batch temperature was monitored with a glass thermometer.

Since the steel thermocouple is expected to undergo a slight corrosion when immersed in the electrolyte, the comparison aimed at evaluating the differences when the probe is substituted with an inert one (i.e. glass thermometer). The current density is here referred to the electrode area; in the main manuscript it was referred to the photovoltaic area. The assembly consisted of two DSA electrodes (Ø = 1.7 cm; inter-electrode gap 3 mm). The electrolyte was 20%wt NaCl solution, heated at 80°C and pumped inside the reactor at a flow rate of 40 mL/min.

The two curves show no significant difference; this proves that the slight corrosion of the stainless has no relevant effect on the electrolysis performance.
